# Comparative Transcriptomic Profiling Reveals Differences in Initiation of Antiviral Response in Low rAAV Producing HEK293 Suspension Cells

**DOI:** 10.1002/biot.70282

**Published:** 2026-07-09

**Authors:** Georg Smesnik, Nikolaus Virgolini, Astrid Dürauer, Nicole Borth

**Affiliations:** ^1^ Department of Biotechnology and Food Science CD Laboratory of Knowledge‐Based Production of Gene Therapy Vectors, Institute of Bioprocess Science and Engineering, BOKU University, Muthgasse Vienna Austria; ^2^ Department of Biotechnology and Food Science Institute of Bioprocess Science and Engineering, BOKU University, Muthgasse Vienna Austria; ^3^ Department of Biotechnology and Food Science Institute of Animal Cell Technology and Systems Biology BOKU University, Muthgasse Vienna Austria

**Keywords:** adeno‐associated virus, cell engineering, gene therapy, productivity, transcriptomics, transient transfection

## Abstract

Recombinant adeno‐associated virus (rAAV) vectors have become the leading platform for in vivo gene delivery, yet their industrial‐scale production remains inefficient and costly. Current manufacturing, which primarily relies on transient triple‐plasmid transfection in HEK293‐derived cells, is limited by low vector yields, high empty‐to‐full capsid ratios, and pronounced batch‐to‐batch variability. To contribute to the understanding of underlying host factors, we compared transcriptomic profiles of five HEK293‐derived producer lines with varying rAAV yields over a 72‐hour production time course. The low‐producing BalCD line showed poor transfection efficiency, reduced plasmid‐derived transcript levels, exclusive interferon expression and a rapid activation of interferon‐stimulated genes (ISG), indicating strong antiviral restriction for vector productivity. In contrast, high‐producing lines mounted a reduced inflammatory and innate immune response with limited induction of antiviral genes, supporting sustained vector production. Expression patterns of high‐producing cell lines further exhibited transcriptional signatures resembling those seen for producer cells arrested in the G0/G1 cell cycle phase, which potentially favors efficient vector genome replication and capsid assembly. The observed differences in the regulation of antiviral and cell cycle–associated pathways highlight key molecular determinants of rAAV productivity and provide a foundation for targeted host engineering strategies aimed at enhancing rAAV manufacturing efficiency.

## Graphical Abstract and Lay Summary

Comparative, time‐resolved transcriptomic profiling of HEK293 producer cell lines identified differences in antiviral signaling and cell cycle regulation between producer phenotypes. Low‐producer exhibited strong interferon‐mediated antiviral responses that may restrict vector production, whereas high‐producers showed reduced innate immune activation and transcriptional signatures associated with G0/G1 arrest, potentially favoring efficient vector assembly.

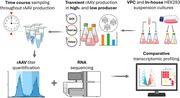

**Abbreviations**
CCNB: cyclin B
CCND: cyclin D
CRKL: CRK‐like proto‐oncogene, adaptor protein
CXCL: C‐X‐C motif chemokine ligand
ddPCR: droplet digital polymerase chain reaction
E1A: early region 1A protein
E1B: early region 1B protein
E2F, E2F: transcription factor
ELISA: enzyme‐linked immunosorbent assay
GADD45: growth arrest and DNA damage‐inducible
GFP: green fluorescent protein
HEK293: human embryonic kidney 293 cells
IFIH: interferon induced with helicase C domain
IFIT: interferon‐induced protein with tetratricopeptide repeats
IFITM: interferon‐induced transmembrane protein
IRF: interferon regulatory factor
ISG: interferon‐stimulated gene
ISGF: interferon‐stimulated gene factor
JAK: Janus kinase
MAVS: mitochondrial antiviral‐signaling protein
MX: MX dynamin‐like GTPase
MYC: MYC proto‐oncogene, bHLH transcription factor
OAS: 2'‐5'‐oligoadenylate synthetase
OASL: 2'‐5'‐oligoadenylate synthetase‐like
rAAV: recombinant adeno‐associated virus
Rep: replication protein
RIG‐I: retinoic acid‐inducible gene I
SFN: stratifin
STAT: signal transducer and activator of transcription
TBK: TANK‐binding kinase
TGFB: transforming growth factor beta
THBS: thrombospondin
TICAM: Toll‐like receptor adaptor molecule
TP: tumor protein
TYK: tyrosine kinase

## 1 Introduction

Adeno‐associated virus vectors (rAAV) based gene therapies have demonstrated remarkable clinical efficacy in recent years [1, 2, 3, 4]. With five rAAV products approved by the U.S. Food and Drug Administration (FDA) since 2021 and more than 280 clinical trials currently ongoing, viral vector‐based gene therapy is a rapidly growing and promising field that offers opportunities for treating a wide variety of previously untreatable genetic disorders [3, 4].

Among viral vectors, rAAVs have emerged as the leading platform for in vivo gene delivery because they can efficiently transduce both dividing and non‑dividing cells [5, 6], and their capsids can be engineered to confer broad and tunable tissue tropism [7, 8, 9]. In addition, rAAVs exhibit a comparatively favorable safety profile, characterized by generally low immunogenicity upon first administration [10, 11, 12, 13, 14] and a predominantly non‑integrating behavior of the vector genome [5, 15]. In contrast to integrating vectors, rAAV genomes largely persist as episomal concatemers within the nucleus, thereby limiting the risk of insertional mutagenesis while enabling long‑term transgene expression in the cell [16, 17, 18, 19]. Despite their clinical success, rAAV‐based gene therapies remain among the most expensive medications on the market [20]. Current production relies mainly on transient triple transfection of rAAV production plasmids in human embryonic kidney cells (HEK293) [21, 22], with substantial costs from raw materials, low vector yields and poor recovery of functional product [23]. High ratios of empty or defective capsids produced by the host cell and batch‐to‐batch inconsistencies further limit manufacturing efficiency and drive the cost of clinical‐grade vector preparations [22, 23, 24].

While natural AAV infection achieves highly efficient replication and capsid assembly in the presence of a helper virus [25, 26], the production restrictions of triple plasmid transfection, where minimal helper factors are provided on a plasmid, remain under extensive investigation using multi‐omics approaches. Recent observations have revealed substantial cell‐to‐cell heterogeneity within rAAV production cultures, with only a minority of transfected cells actively assembling capsids [27, 28]. In addition to cell cycle regulation determining host efficiency [28], endoplasmic reticulum (ER) stress, and unfolded protein response signaling [29, 30], chromatin remodeling gene regulation [31, 32], proteasomal viral protein degradation [33], and most prominent, robust immune‐ and inflammatory responses to plasmid transfection and viral particles themselves have all been implicated in limiting rAAV yields in transient triple transfection systems [30, 31, 32, 34]. Given the diverse cellular responses observed across HEK293 variants and production systems, we aimed to systematically characterize and compare the transcriptomic profiles of five distinct rAAV‐producing cell lines with differing production capacities, with the goal of identifying key transcriptional differences underlying titer variation and uncovering potential engineering targets to optimize rAAV production in these systems.

In this study, we present a comparative transcriptome analysis, including the industrially relevant Virus Production Cell Line (VPC) platform, optimized for viral vector production, alongside with two internally generated suspension cell lines [35]. We compared the low rAAV‐producing internal cell line BalCD against the four high producing ones, including three VPC derived lineages adapted to the recommended VPC medium or adapted to Hyclone peak expression medium (PE) or BalanCD HEK293 medium (BalCD) named VPC_VPC, VPC_PE and VPC_BalCD, alongside the in‐house generated PE cell line [35]. Changes in gene expression patterns were analyzed across a 72‐hour time course post‐transfection, capturing dynamic gene expression changes throughout production.

The high producing cell lines, irrespective of their different origin, revealed shared, cell line‐ and medium‐specific patterns. The resulting gene sets were subjected to pathway and functional enrichment analysis to explore their biological relevance and potential mechanistic contributions to differential rAAV productivity across cell lines.

## 2 Results and Discussion

### 2.1 Experimental Design

Transient rAAV production followed a standardized triple‐plasmid transfection protocol across all five cell lines in shake‐flask scale. Cultures were sampled at seven timepoints: pre‐transfection, and 6, 18, 24, 30, 48 and 72 h post‐transfection. These sampling timepoints were selected to provide high temporal resolution across the full production phase, covering early host cell responses after the transfection process and subsequent viral component synthesis, as well as the critical window of rAAV packaging and intracellular vector accumulation previously reported [21, 31, 36]. This design was chosen to allow close examination of gene expression dynamics during the major phases of transient viral vector production: early response to transfection (peaking at 6 h), onset of prime rAAV gene expression, capsid assembly and packaging (12 – 32 h), followed by peak rAAV production and cell response (48 – 72 h).

For standardized production conditions, four biological replicates were conducted for each of the five cell lines, all transfected with pHelper, pRepCap, and pGOI plasmids.

### 2.2 Differences in the Transcriptome Signature and Response Pattern in the Low Producing Cell Line BalCD

**FIGURE 1:**
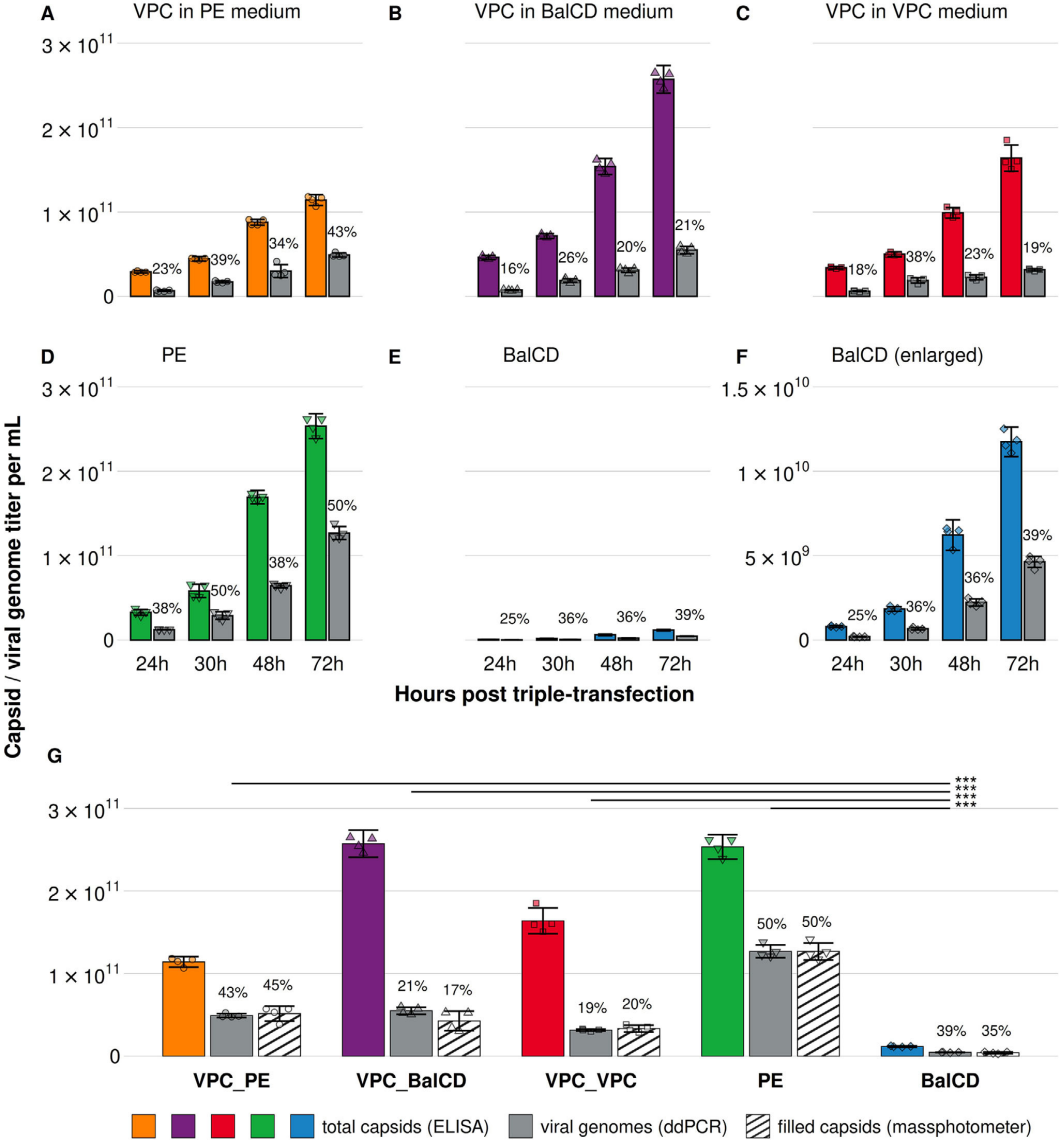
Total viral capsid‐ and genome titers over time. (A–E) Volumetric titers of total capsids (colored bars; ELISA) and viral genomes (gray bars; ddPCR) per mL at indicated time points post‐transfection for five conditions with filled‐to‐total rAAV ratio expressed in percentage. Error bars represent standard deviation among four biological replicates (shaped points). Panel (F) displays low‐producing BalCD on a reduced scale to visualize production kinetics over time. (G) Comparison of final 72 h titers with mass photometry confirmation of filled‐to‐total ratios (striped bars). Volumetric titers shown for mass photometry were calculated based on the full capsid fraction measured by mass photometry, multiplied by the total capsid titer determined by ELISA. Bars and asterisks indicate significant differences by one‐way ANOVA with Dunnett's post hoc test (p‐adj: *** <0.001, ** <0.01, * <0.05) in viral genome‐ and capsid titers of each condition compared to BalCD. Mass photometry measurements are detailed in the Figure S2.

Following transfection, all cell lines exhibited comparable growth kinetics. Starting from an initial density of 2 × 10^6^ cells/mL at the time of transfection, cultures grew to final densities ranging from 4.1 ± 0.45 × 10^6^ cells/mL for VPC_VPC to 5.13 ± 0.14 × 10^6^ cells/mL for VPC_BalCD. Cell viability remained consistently high across all lines and did not fall below 87% throughout the production phase (Figure S1A). This was consistent with previous internal production runs and comparable studies [29, 30, 31]. Figure 1 presents the volumetric titers of viral genomes and capsids, quantified from 24 h post‐transfection. Titers increased progressively over time, showing near‐exponential accumulation before reaching peak level after 72 h. The PE cell line achieved the highest overall yields, with maximal titers of 2.53 ± 0.15 × 10^1^ ^1^ capsids/mL and 1.27 ± 0.08 × 10^1^ ^1^ viral genomes (vg)/mL, corresponding to a filled‐to‐total capsid ratio of approximately 50% (Figure 1D). In contrast, BalCD produced significantly lower titers, reaching only 1.17 ± 0.09 × 10^10^ capsids/mL and 4.63 ± 0.03 × 10^9^ vg/mL, resulting in a filled capsid fraction of 39% (Figures 1E,F). All VPC‐derived cell lines significantly outperformed BalCD in vector yield (Figures 1A–C). Among them, VPC_BalCD revealed the highest productivity, with final titers of 2.57 ± 0.16 × 10^1^ ^1^ capsids/mL and 5.50 ± 0.43 × 10^10^ vg/mL (Figure 1B). Relative quantification of viral genomes and capsids measured by standard analytical methods (ddPCR, ELISA) was complemented by mass photometry (MP) analysis (Figures 1G and S2). MP measurements performed on further purified samples allowed discrimination of rAAV species into filled, partially filled, and empty particles based on particle mass (Figure S2). The resulting filled‐to‐total particle ratios were used to calculate volumetric filled‐particle titers from the total capsid concentrations determined by ELISA (Figure 1G).

Transfection efficiency was assessed based on the fraction of GFP‐expressing cells at 24, 48, and 72 h post‐transfection. At 24 h, PE and all three VPC‐derived cell lines exhibited moderate to high transfection rates, with GFP‐positive fractions ranging from approximately 38% in PE to 50%–55% in the VPC lines, respectively. In contrast, BalCD showed a markedly lower transfection efficiency, with only ∼12% GFP‐positive cells. Along with the reduced fraction of positive cells, BalCD also displayed lower fluorescence intensity per cell, indicating weaker overall transgene expression (Figure S3A). The fraction of GFP‐positive cells increased slightly between 24 and 48 h post‐transfection and remained stable or decreased marginally until 72 h. PE and VPC cell lines reached ∼55%–65% GFP‐positive cells, whereas BalCD remained below 20% throughout. Overall, BalCD consistently showed lower transfection efficiency and expression levels compared to PE and VPC‐derived lines (Figure S3B), while transfection rates of higher‐producing cell lines were within ranges reported elsewhere [27]. Although phenotypic characteristics such as growth and viability during cell line cultivation prior to the rAAV production experiment did not indicate any apparent cellular stress (Figure S1B), the reduced transfection efficiency and lower GFP expression intensity, might suggest the presence of a cell line‐specific pattern limiting entry and/or intracellular processing of transfection complexes. Altered basal gene expression profiles, in particular upregulated interferon (IFN) type I and II signaling pathways, have previously been described for low‐producing HEK293 cells and may interfere with efficient transfection and initiation of viral particle synthesis [34]. However, gene set enrichment analysis (GSEA) based on the molecular signatures database (MSigDB) and pairwise comparisons between the low‐producing BalCD and the four high‐producing cell lines pre‐transfection revealed no enrichment of IFN type I signaling. IFN type II (IFN‐γ) was slightly positively enriched (Table S1), driven by elevated expression of selected IFN‐responsive genes, in the absence of detectable IFN type I or type II expression. Among the upregulated genes were interferon‐induced protein 44 (IFI44) and interferon‐induced protein 44‐like (IFI44L), both of which have been reported to interfere with viral infection at early stages in overexpression studies with respiratory syncytial virus (RSV) infection [32, 37]. While the functional relevance of elevated IFN‐responsive genes for rAAV production remains to be determined, they may reflect differences in antiviral response regulation between low‐ and high‐producing cell lines. Besides IFN‐related pathways, GSEA at 0 h also identified enrichment of HEDGEHOG signaling, epithelial–mesenchymal transition (EMT), and KRAS signaling downregulated gene sets, reflecting broader basal transcriptional differences between these cell lines (Table S1). In particular, the EMT gene set includes genes associated with altered cytoskeletal organization, membrane dynamics, and intracellular trafficking processes, which might influence transfectability [38, 39].

Besides sufficient intracellular availability of plasmid genes after transfection, efficient rAAV production depends on cytoplasmic transport and nuclear entry [36]. Previous studies demonstrated impairments in both plasmid uptake and nuclear delivery in low‐producing cells, resulting in plasmid‐independent reductions in expression levels of delivered genes [40]. In the present study, plasmid‐derived gene expression in BalCD followed a comparable trend, showing the lowest transfection efficiency (Figure S3) and subsequently reduced transcript levels for all plasmid‐derived genes compared to the high‐producing cell lines (Figure S4B). However, despite differences in absolute transcript abundance, expression dynamics over time remained highly correlated (Spearman's ρ > 0.79) between all cell lines and plasmids (Figure S4A), indicating no apparent selective transcriptional impairment of individual plasmid‐derived features. Together, these observations point toward limitations in the transfection process, potentially associated with pre‐existing molecular signatures in BalCD cells.

### 2.3 Global Transcriptome Comparison Reveals Cell Line Specific Signatures Independent of Growth Medium Used

**FIGURE 2:**
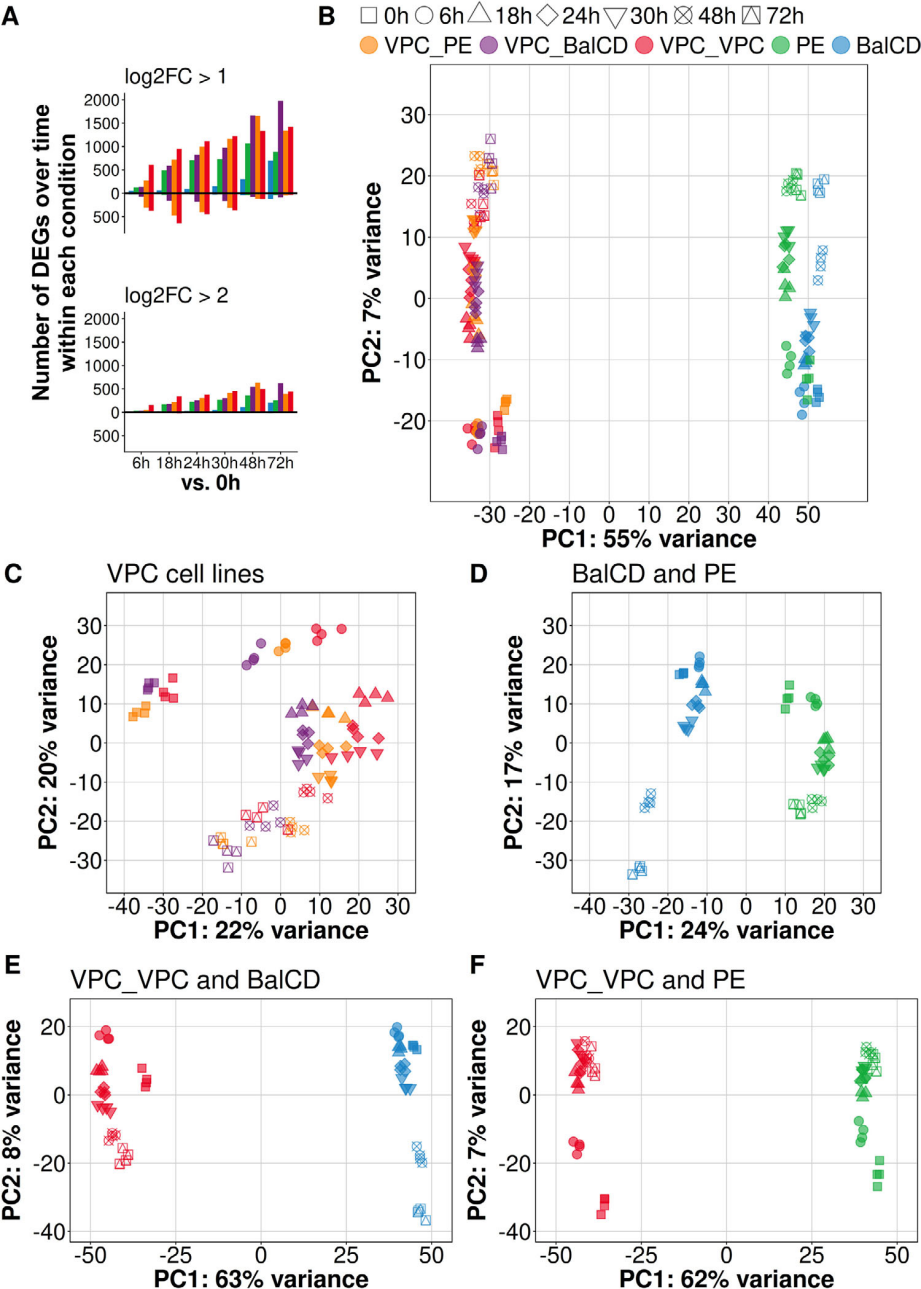
Global transcriptional differences across conditions. (A) Number of differentially expressed genes (DEGs) categorized by their log2FC relative to timepoint 0 h (prior to transfection) within each condition. Conditions are indicated by the corresponding color. The number of upregulated genes at each time point is shown as bars above the baseline (log2FC: 0), the number of downregulated genes is shown as bars below the baseline. Genes were considered differentially expressed at an adjusted p‐value threshold <0.05 and were categorized according to log2FC thresholds of >1 (upper panel) and >2 (lower panel). (B‐F) Principal component analysis (PCA) plots of (B) full dataset including five conditions; (C) VPC‐derived cell lines; (D) adaptation‐derived cell lines (BalCD, PE); (E) original VPC_VPC and BalCD (low producer); (F) original VPC_VPC and PE (high producer). Each point represents one biological replicate, shapes indicate the timepoint and colors correspond to conditions.

To assess global transcriptional response to plasmid transfection and viral production, transcriptome data were examined for differentially expressed genes (DEGs) within each cell line by comparing the respective baseline expression prior to transfection (0 h), with that at subsequent post‐transfection sampling time points between 6 and 72 h. DEGs were identified using a significance threshold of adjusted *p*‐value < 0.05 and further categorized by magnitude using log2FC thresholds of >1 and >2. The temporal distribution of these DEGs is shown in Figure 2A, where the number of up‐ and down‐regulated genes at each timepoint is shown for both log2FC categories. As the experimental design was focused on comparing production‐related transcriptional responses across different producer phenotypes and media compositions over time, no separate mock‐transfected controls were included. Instead, the 0 h pre‐transfection samples served as internal reference for each cell line. Across all cell lines, DEG numbers increased over time following transfection, with the highest numbers at later timepoints, indicating a progressive and sustained response to the rAAV production procedure. Applying the more stringent cutoff (log2FC >2) reduced identified DEG counts but preserved the overall temporal trend as previously reported [31]. Compared to the other cell lines, BalCD consistently exhibited lower DEG numbers across all time points, whereas PE displayed transcriptional dynamics more similar in counts and progression to the VPC lines (Figure 2A).

Principal component analysis (PCA) of all expressed genes passing the low‐count filter (20,697 genes) revealed that host cell background was the primary source of transcriptional variance (Figure 2B). The first principal component (PC1) explained 55% of variance and separated VPC‐derived cell lines from each of the two adaptation‐derived PE and BalCD cells. The second principal component (PC2) explained 7% of variance and reflected temporal transcriptional changes following transfection, separating early from later production phases. While this temporal progression was observed across all cell lines, BalCD showed a comparatively distinct distribution along PC2, suggesting different transcriptional dynamics over time. Within the VPC‐derived group, all cell lines clustered closely despite cultivation in different media, indicating only minor transcriptional differences associated with media adaptation (Figure 2C). In contrast, PE and BalCD formed clearly separate clusters without direct overlap, indicating a more fundamental transcriptional divergence between these cell lines derived by adaptation directly from the adherent parent (Figure 2D). Comparisons of VPC_VPC with BalCD (Figure 2E) and PE (Figure 2F) further confirmed that cell line‐specific differences dominated the transcriptional landscape, with PC1 explaining 63% and 62% of the variance, respectively. Together, these results demonstrate that cell line identity is the primary determinant of global transcriptional signatures, whereas transfection and/or vector production induce an additional time‐dependent response, consistent with previous comparative transcriptomic studies [29, 30].

### 2.4 Expression Trajectories of Top Differentially Expressed Genes

**FIGURE 3:**
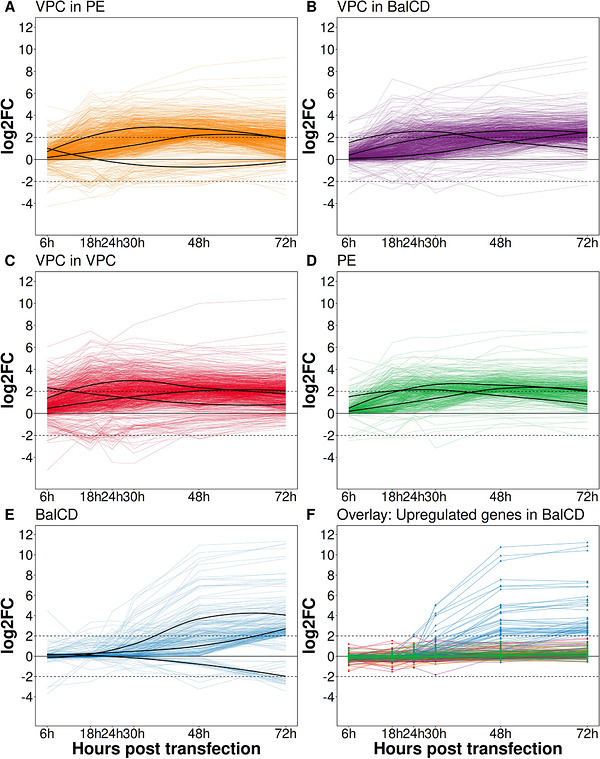
Gene expression trajectories over time. (A–E) Trajectories of differentially expressed genes within each condition (p‐adj. <0.05) reaching a log2FC threshold of <2 at any time throughout the production period. Represented as line plots showing log2FC relative to baseline expression at 0 h (pre‐transfection) across the production time course. Solid black lines denote centroids of three discrete trajectory clusters calculated based on Pearson correlation distance with applied Ward's minimum variance method. (F) Overlay of expression trajectories for a gene subset exclusively and highly differentially expressed in BalCD compared with each other cell line, with colors corresponding to conditions as in panels (A–E).

Analysis of PCA loadings revealed that strong gene upregulation shortly after transfection represented a dominant source of variance across all cell lines. Among the high‐producing cell lines (VPC_VPC, VPC_PE, VPC_BalCD, and PE), genes strongly regulated during production (absolute log2FC > 2 at any time during production) showed highly similar temporal expression patterns and regulation magnitudes (Figure 3A–D). In contrast, the low‐producing BalCD cell line displayed a distinct transcriptional profile. Although fewer genes reached this threshold in BalCD (223 genes) compared to the high‐producing lines (458‐831 genes), BalCD exhibited a pronounced wave of rapid gene induction between 24 and 30 h post‐transfection, reaching log2FC values of up to 12 (Figure 3E). This period (24‐30 h) is associated with the expected onset of rAAV packaging and capsid assembly, following initial viral genome transcription and capsid protein synthesis [21, 31, 36]. While the observed shift may reflect both transfection induced stress and responses to viral components, the delayed response pattern in BalCD aligns well with a reported secondary transcriptional wave linked to viral synthesis [21, 31].

To identify shared and time‐dependent expression patterns, highly up‐regulated genes were clustered based on the similarity of their temporal trajectories using Pearson correlation. Three distinct trajectory clusters were generated based on the individual gene expression trajectories (Figure 3A to E): an early‐response cluster (cluster 1), a mid‐to‐late response cluster (cluster 2), and a small cluster of downregulated genes (cluster 3; Figure S5). Gene ontology and pathway enrichment analysis revealed a broad overlap of biological functions associated with cluster 1 across all high‐producing cell lines (Figure S5), particularly for inflammatory response, cytokine signaling, morphogenesis‐related processes, and motor proteins‐associated functions (Figure S5). These findings are consistent with previous transcriptomic studies, reporting activation of innate immune signaling and inflammatory pathways during rAAV production in various experimental settings [30, 31, 32, 34, 41]. Chung and co‐workers described distinct phases of transcriptional responses where the upregulation of genes related to an inflammatory response peaked around 12 h post‐transfection and remained high until the end of production. Cluster 1 in the present analysis follows a comparable trajectory and confirms the upregulation of inflammatory mechanisms with similar transcriptomic behavior over time. Notably, in contrast to previous observations, enrichment of interferon‐related pathways, following immune‐related signaling as a cellular defense mechanism, was not detected in high‐producing cells within the time of observation [31]. Interestingly, detailed comparative analysis identified a subset of 43 genes strongly induced exclusively in BalCD (Figure 3F). These genes were differentially expressed both over time (compared with 0 h) and in pairwise comparisons against all other cell lines and consisted mainly of interferon stimulated genes (ISG), representing a distinct antiviral transcriptional signature in BalCD.

**FIGURE 4:**
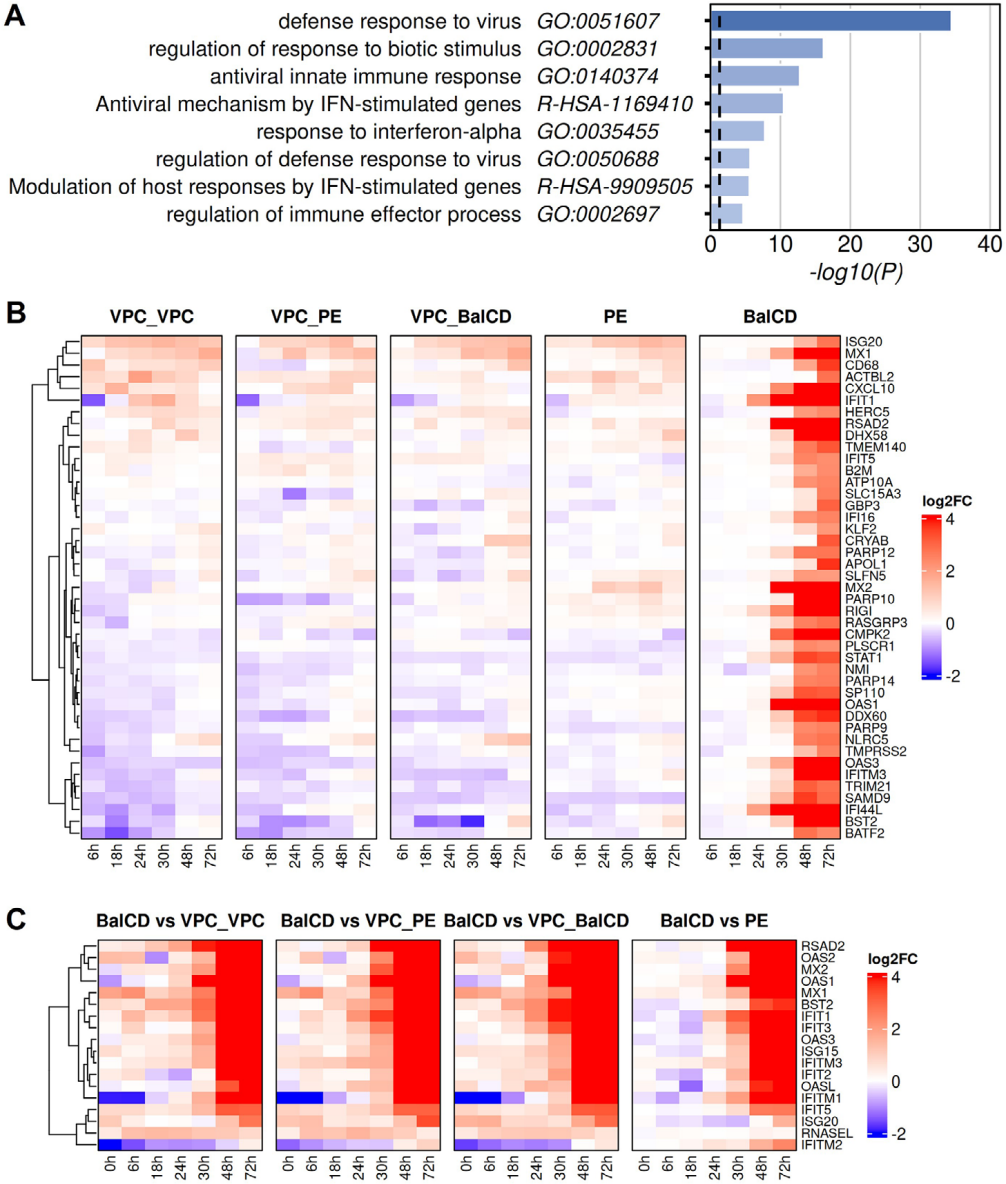
Transcriptomic profiles of BalCD‐specific responses. (A) Gene ontology (GO) and pathway enrichment analysis of genes identified as significant exclusively in BalCD via B‐spline analysis. Bar length represents the ‐log10 p‐value, the vertical dashed line indicates the significance threshold. (B) Heatmaps illustrating time‐course expression profiles (log2FC relative to 0 h) for highly upregulated genes in BalCD across all conditions as shown in Figure 3F. (C) Differential expression heatmaps for antiviral effector genes, showing pairwise comparisons of each condition relative to BalCD. For (B) and (C), row‐wise hierarchical clustering of left panel was used to determine gene order, gene symbols are annotated on the right. Color scale represents log2FC ranging from ‐2 (blue, decreased) to 4 (red, increased), with white indicating no change.

To complement the trajectory clustering analyses, transcriptional dynamics were additionally analyzed using B‐spline regression implemented in the SplineOmics R package [42] (Figure S6A). Subsets of genes either shared by the high‐producing lines (VPC_VPC, VPC_PE, VPC_BalCD, PE) or exclusive to the low‐producing BalCD were then subjected to gene ontology and pathway enrichment analysis. Consistent with the clustering results, high‐producing cell lines shared enrichment of inflammatory response, motor protein, and cell projection‐associated pathways (Figure S6B and S5). In contrast, BalCD‐specific splines (272), revealed strong enrichment for viral defense and interferon‐mediated antiviral mechanisms, further supporting selective activation of ISG‐associated responses in this cell line (Figures 4A and S6B) [43].

### 2.5 Differential Initiation of Antiviral Effector Expression

As Figure 4B highlights the identified subset of highly differentially expressed genes in BalCD. Of the 43 genes identified as uniquely induced in BalCD, 29 were associated with the Gene Ontology term defense response to virus (GO:0051607) and represented ISG (Figure 4B). Most of these genes showed rapid induction 24, 30 or at latest 48 h post‐transfection. While this response could not be explicitly discriminated from overall transfection‐associated effects, the timing suggested a secondary response and was in line with expression kinetics reported in previous mock‐controlled studies. In particular, IFIT2, IFIT3 and OASL, potent anti‐viral effector genes, were upregulated only in triple‐transfected cells, following similar kinetics with a sharp increase at 24 h post‐transfection [34]. Furthermore, multi‐omics analyses comparing mock‐ and AAV‐transfected conditions showed stronger activation of immune‐ and host defense pathways, in AAV transfected cells [41]. Together, these findings suggest that expression of viral defense genes, in addition to reduced transfectability, may contribute to the lower productivity observed in BalCD. This is further explored by a detailed examination of the specific genes involved in these pathways and their respective transcriptional shifts.

**FIGURE 5:**
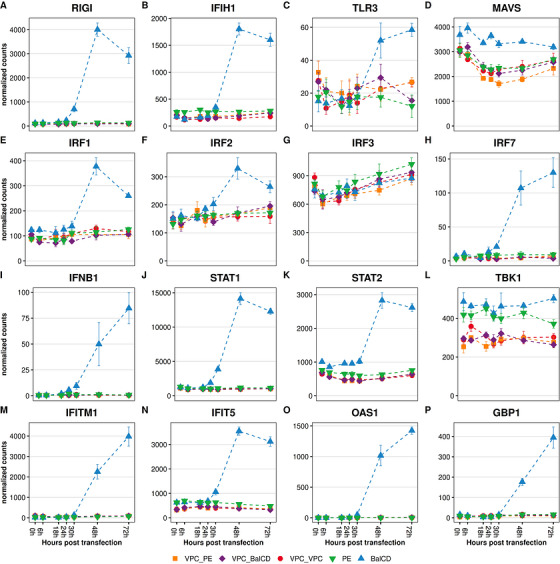
Kinetic profiles of selected genes. (A–P) Data points represent the mean of normalized counts (median‐of‐ratios method) at each indicted time point, with colors and shapes corresponding to conditions. Error bars indicate standard deviation among four biological replicates. Dashed lines illustrate expression trajectories over time course. Gene symbols are annotated on top of each panel.

Among the major antiviral sensing pathways, the RIG‐I–like receptor (RLR) pathway represents a central intracellular viral detection mechanism, with RIG‐I and IFIH1 recognizing cytosolic viral RNA [43, 44]. In the context of rAAV genomes, RNA polymerase III (Pol III)‐mediated transcription of viral DNA into RNA provides an alternative route for RLR pathway activation [45, 46]. The exclusive overexpression of RIG‐I and IFIH1 in BalCD strongly suggests activation of RLR signaling after 24 h (Figures 5A,B).

Recognition of foreign nucleic acids through RLRs promotes activation of MAVS and recruitment of TBK1, which in turn phosphorylates IRF3 and IRF7 [43]. While IRF3 expression levels were comparable across all cell lines, IRF7 transcript levels increased in BalCD from 24 h onwards (Figure 5G,H). Phosphorylated IRF3–IRF7 heterodimers translocate to the nucleus and induce the production of type I interferons (IFN‐I). As the central mediators of this response, these interferons drive the initiation of downstream antiviral effector gene expression via canonical or non‐canonical IFN‐signaling pathways. The observation that interferon‐beta 1 (IFNB1) induction was unique to BalCD, following the same activation pattern as other pathway components, may explain the absence of similar antiviral responses in high‐producing cells. (Figure 5I).

A second route for IFN‐I induction involves Toll‐like receptors. Although many TLRs were considered weakly expressed or absent in HEK293 cells, TLR2, and TLR3 have previously been linked to ISG induction during rAAV production [30, 31, 47]. In the current study, TLR3 expression increased rapidly in BalCD between 30 and 48 h post‐transfection, whereas only moderate induction was observed in VPC and PE cells. (Figure 5C, Table S2). Slightly elevated transcript levels of downstream adaptor TICAM1 further support enhanced activation of this pathway in BalCD, leading to TBK1 and IRF7 activation and ultimately IFN‐I production [43, 47].

Following the canonical type I interferon (IFN‐I) pathway, secreted IFN‐I leads to activation of the JAK–STAT signaling cascade. Phosphorylated STAT1 and STAT2 form a complex with IRF9, generating the heterotrimeric transcription factor ISGF3, which binds to interferon‐stimulated response elements (ISRE) in the promoters of ISG and promotes their transcription [48, 49]. Interestingly, although STAT1 and STAT2 exhibited similar expression kinetics (Figures 5J,K, Table S2), IRF9 transcripts were not detected in any sample. Despite this, clear transcriptional activation of ISRE‐dependent ISGs, including members of the IFIT family, MX1/2, OAS1/2, ISG15, and CXCL10 was observed in BalCD (Figure 4B). This suggests that the lack of IRF9 may be compensated through non‐canonical mechanisms of ISG activation. Potential alternatives include direct regulation by elevated levels of IRF1, IRF2, or IRF3 (Figure 5E–G), signaling via STAT5–CRKL complexes, or STAT1 homodimer formation (Table S2) [43, 50].

Many of the induced ISGs encode potent antiviral effectors that suppress viral replication, protein synthesis, or intracellular transport [51, 52, 53, 54, 55, 56]. Induction of members of the oligoadenylate synthetase (OAS) family (OAS1, OAS2, OAS3, and OASL) together with RNase L suggests activation of RNA degradation pathways capable of broadly reducing cellular biosynthesis (Figures 4B,C, and 5O and Table S1) [52, 53]. Similarly, IFIT and IFITM proteins (IFIT1, IFIT2, IFIT3, IFIT5, IFITM1), interfere with viral RNA recognition, replication, and protein synthesis (Figure 4C and Table S2) [56], whereas MX proteins and ISG15 inhibit intracellular viral trafficking and assembly [51, 54, 56, 57, 58, 59]. MX proteins are localized in the cytoplasm and nucleus, enabling effective targeting of viruses with a nuclear replication phase, such as AAVs. Cytoplasmic MX1 restricts viral replication by trapping viral components within ring‐like oligomers, whereas MX2 interferes with viral infection at the nuclear pore complex by inhibiting nuclear entry [59, 60]. ISG15 covalently modifies viral proteins through ISGylation and thereby disrupts viral assembly and replication [54]. These antiviral functions are consistent with impaired vector production in BalCD. Supporting their functional relevance, knockout of OAS1 in lentiviral HEK293 production systems increased vector yields by almost seven‐fold [61].

A potential reduction of any negative impact of antiviral signaling on rAAV productivity has also been demonstrated through the use of small molecule inhibitors and genetic modulation of innate immune pathways. Inhibition of PRR‐associated signaling (MRT67307, BX795, MRT68844) or JAK/STAT signaling (Tofacitinib, AZD1480, CYT387, TG101348, Ruxolitinib) improved rAAV yields in multiple studies [34, 41, 62, 63]. In particular, TBK1 inhibition by MRT67307 suppressed ISG induction and enhanced vector production, whereas artificial activation of PRR signaling using nucleic acid agonists reduced rAAV titers [41]. Similarly, inhibition of JAK1/TYK2 signaling by Ruxolitinib increased rAAV production without negatively affecting full‐to‐empty ratios [34]. Extending such inhibitory approaches through gene editing enabled the generation of STAT1‐knockout clones with slightly improved capsid quality and maintained vector titers [64]. Thus, further refinement of gene editing strategies targeting these cellular pathways may provide opportunities for future cell line engineering and process optimization.

### 2.6 E1A and E1B Expression and Cell Cycle‐associated Signatures

**FIGURE 6:**
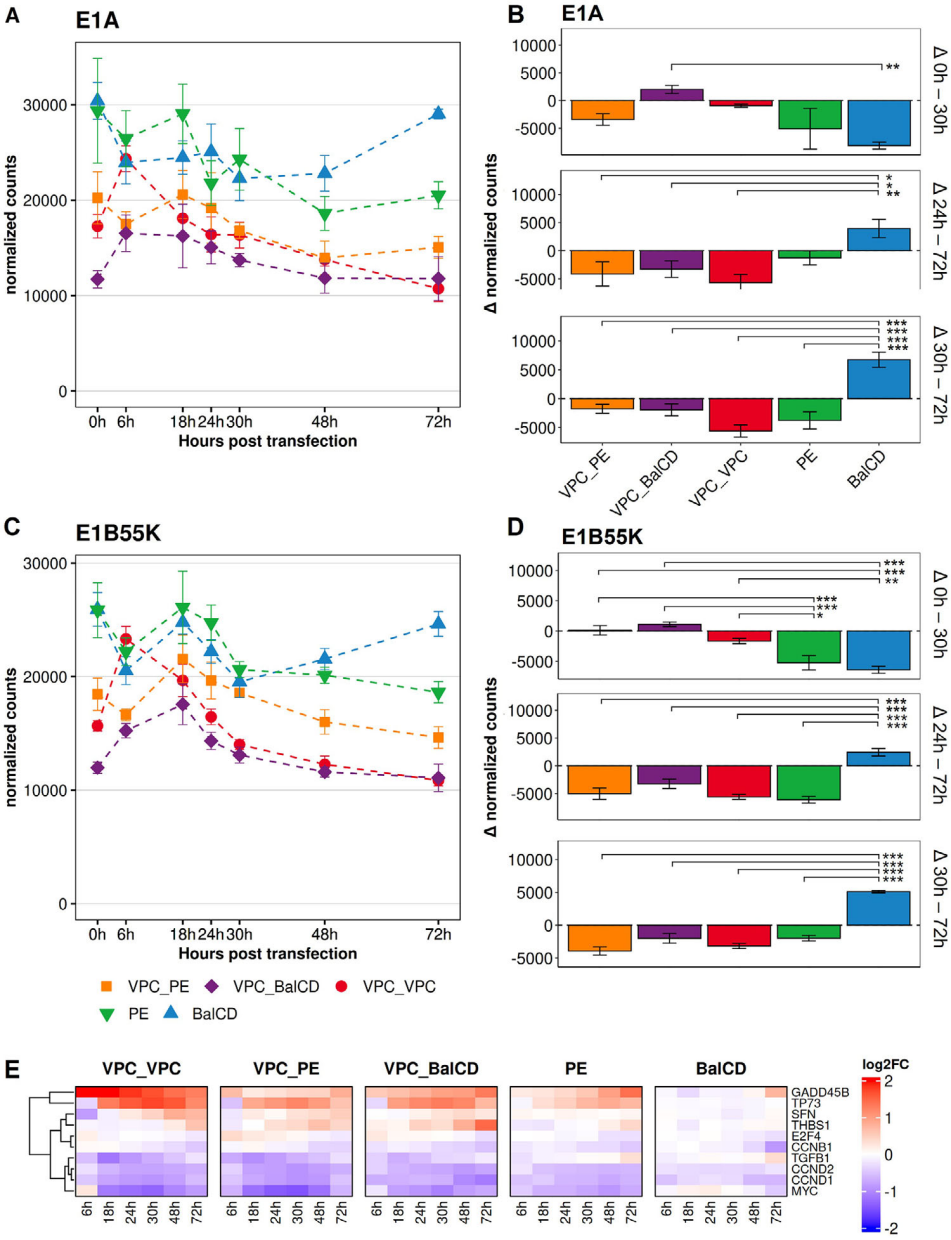
Kinetic profiles and gene expression trends of E1A, E1B55K and cell cycle related genes. Data points in line plots of panel (A) and (C) represent the mean of normalized counts (median‐of‐ratios method) at each indicted time point, with colors and shapes corresponding to conditions. Dashed lines illustrate expression trajectories over time course. Bar plot in (B) and (D) show rates of change (Δ normalized counts) for three time intervals within the production period (Δ 0 – 30 h; Δ 24 – 72 h, Δ 30 – 72 h). Error bars indicate standard deviation among four biological replicates. Horizontal lines and asterisks indicate significant differences in expression trends between BalCD and high‐producing conditions, determined by one‐way ANOVA with Tukey's post hoc test (p‐adj: *** <0.001, ** <0.01, * <0.05) on calculated rates of changes. Gene symbols are annotated on top of each panel. (E) Heatmaps illustrating time‐course expression profiles (log2FC relative to 0 h) for p53 and cell cycle related genes across all conditions.

In addition to the inflammatory and antiviral host cell responses demonstrated here, and to varying extent reported across different experimental approaches in previous studies [30, 31, 32, 34, 41], recent observations linked transcriptomic signatures of producer cells with distinct cell cycle regulation stages. Considering the critical role of the helper function of integrated adenoviral E1A and E1B in cell cycle regulation of HEK293 cells, as well as their interference with plasmid‐derived Rep 78 protein during rAAV production, the cell cycle state of producer cells has been shown to play an important role in transient rAAV production [28, 65, 66, 67]. Improved rAAV assembly has been associated with cells arrested in cell cycle progression, predominantly in the G0/G1 phase [28, 66, 67, 68, 69]. In these producing cell populations, reduced transcript levels of E1A and E1B (E1B55K), potentially due to interaction with the AAV Rep78 protein were demonstrated in mock‐controlled experiments 24, 48 and 72 h post‐transfection [28, 69]. In contrast to previous observations in comparative studies between low‐ and high‐rAAV producing HEK293 cells [40], we also observed elevated E1A and E1B55K transcript levels in BalCD throughout the production period (Figure 6A,C and S7A,B). While the low‐producer BalCD exhibited relatively high levels of both genes already at timepoint 0 h, which remained high throughout production (Figures 6A,C and S7A,B), the transcriptional dynamics were different in high‐producers (Figures 6A,C). While non‐uniform effects of up‐ and downregulation occurred to varying extents in all five cell lines directly after transfection, BalCD exhibited an increasing expression trend from 24 h onward, in contrast to the other cell lines. This is reflected in the rate of change of normalized counts across three‐time intervals, representing changes directly after transfection (0–30 h) and during later production phases (24–72 h and 30–72 h; Figure 6B and D). Although most cell lines returned to their respective basal expression levels at 72 h, the temporal dynamics differed between BalCD and high‐producing cells, which may point toward differences in regulation or interactions affecting helper gene expression during rAAV production. Furthermore, in agreement with findings by Mullick et al., downregulation of peroxisome‐ and lysosome‐associated genes was observed, indicative of altered cell proliferation dynamics (Figure S8) [70, 71]. Moreover, several genes associated with G0/G1 cell cycle arrest in producing cells showed similar regulation as high‐producers in our study. These included the p53 pathway‐related tumor suppressor TP73 [72], the apoptosis inducer SFN and the growth arrest associated gene GADD45B, which were upregulated in higher‐producing cell lines (Figure 6E). In parallel, increased expression of THBS1, a negative regulator of cell cycle progression [73], was observed, alongside downregulation of key drivers of cell cycle progression, including MYC, E2F4, CCND1, CCND2, and CCNB1 (Figure 6E). Collectively, the comparison of previously identified transcriptional signatures for triple‐transfected producing‐ or nonproducing cell populations revealed transcriptomic similarities with high‐producing and low‐producing cell pools in our present study. This might suggest that a high proportion of cells in VPC‐derived‐ and PE cells reside in G0/G1 phase, a state that has been correlated with increased rAAV assembly [28].

## 3 Concluding Remarks

In summary, our results demonstrate that low rAAV productivity in our BalCD‐adapted cell line is associated with poor transfection efficiency, reduced transcript levels of transfected genes and a pronounced antiviral response characterized by the exclusive expression of interferon‐β and a rapid induction of ISG, likely impairing viral synthesis through various antiviral effects. While the absence of a mock‐transfected control for each individual condition prevents an explicit discrimination between effects caused by the transfection procedure itself from direct responses to viral particle synthesis, a comparison of basal expression levels, alignment of results with previously reported mock‐controlled datasets, and the distinct temporal response pattern together strongly support the interpretation that the observed patterns in BalCD are directly linked to lower productivity. Nevertheless, further experimental validation is required to confirm the causative nature of these transcriptional trends.

In contrast, despite pronounced differences in the global transcriptional profiles of VPC‐derived and PE high‐producing cell lines, they shared a reduced inflammatory and innate immune response to the production process, characterized by the absence of interferons and the avoidance of strong antiviral effector gene activation. Moreover, these cell lines, despite their different origins, exhibited transcriptional signatures similar to those of reported producer cells arrested in the G0/G1 cell cycle phase, potentially driven by interactions between Rep78 and integrated helper genes E1A and E1B. Together, the findings from this comparative analysis of industrially relevant VPC‐derived cell lines cultivated in various media formulations and optimized for rAAV production with our in‐house‐generated suspension producer cell lines are consistent with previous observations in low‐producing HEK293 cells regarding impaired transfectability and increased antiviral activation. Thus, this study underscores the important role of host antiviral signaling and cell cycle‐associated processes in influencing rAAV productivity.

## 4 Material and Methods

### 4.1 Cell Lines and Cultivation

HEK293 cells from two distinct sources were utilized. Commercially available Virus Production Cells 2.0 (Gibco, Thermo Fisher Scientific, named VPC) were cultivated in the original Viral Production Medium (Gibco, Thermo Fisher Scientific), supplemented with 4 mM GlutaMAX (Gibco, Thermo Fisher Scientific). In addition two independent suspension‐adapted HEK293 cell lines, generated by direct adaptation from adherent cultures (ATCC‐CRL‐1573) to serum‐free suspension conditions, as described in our previous work were used and cultivated in BalanCD HEK293 medium (Fujifilm, named BalCD) and HyClone peak expression medium (Cytiva, named PE), respectively [35]. VPC were adapted to both BalanCD HEK293 medium (Fujifim) and HyClone peak expression medium (Cytiva) serving as media control. BalanCD HEK293 medium (Fujifilm) was supplemented with 4 mM L‐glutamine (Sigma‐Aldrich) and 0.1% Poloxamer 188 (Gibco, Thermo Fisher Scientific). All cultures were initiated in parallel from cryopreserved stocks and maintained in 125 mL Erlenmeyer flasks (Corning) using a working volume of 25 mL until expansion. Cells were cultivated at 37°C in a humidified atmosphere with 5% CO_2_ under agitation at 220 rpm in a Climo‐Shaker ISF4‐X incubator (Kuhner).

Cell cultures were expanded through a series of sequential passages using Erlenmeyer flaks with vent cap (Corning) of increasing capacity (125, 250, 1000, 3000 mL) to achieve the required cell count. Starting from an initial working volume of 25 mL, cultures were scaled up through 65, 250 mL up to a final working volume of 600 mL per cell line, reaching a target cell density of 2 × 10^6^ cells/mL. Expanded cultures were subdivided into four independent replicates per cell line in 250 mL Erlenmeyer flasks with vent cap (Corning) and diluted with fresh media to a starting working volume of 75 mL, maintaining a viable cell density of 2 × 10^6^ cells/mL prior to transfection. Viable cell density was determined using the trypan blue exclusion method with an automated ViCell XR (Beckman Colter) device.

### 4.2 Transfection Process

Prepared cultures were transfected with a transfection cocktail of 5% (v/v) of the final working volume, containing PBS, PEI PRO (Polysciences) as transfection reagent and plasmid DNA of three plasmids. All three plasmids, pGOI (ITR.CMV.GFP.Luc.ITR), pHelper (E2A.E4.VA) and pRepCap (AAV8) were provided by our partner Boehringer Ingelheim International. In detail, a plasmid solution and a transfection reagent solution were prepared separately, each diluted in half the final volume of PBS. The plasmids were combined at a total concentration of 1 µg/mL and a molar ratio of 2:2:1 (pGOI:pRepCap:pHelper). The second solution contained the transfection reagent at 2 times the total DNA amount. Both solutions were gently mixed and incubated 10 min at room temperature before they were carefully combined and incubated for another 15 min at room temperature to allow complex formation. Next, the complex solution was added dropwise to the culture replicates.

### 4.3 Transient rAAV Production, in Process Sampling and Harvest

rAAV production was carried out for 72 h, during which cultures were sampled at 6, 18, 24, 30, 48, and 72 h post transfection to determine rAAV titers, assess growth rates and culture viability, and collect samples for total RNA sequencing. Total RNA samples were centrifuged at 300 g for 5 min, resuspended in 1x DNA/RNA shield (Zymo Research), and stored at −80°C, along with titer samples, until further processing. In addition, transfection efficiency was estimated by quantifying GFP‐positive cells at 24, 48 and 72 h post transfection by flow cytometry using a CytoFLEX S (Beckman Coulter). For analysis, samples were centrifuged at 300 g for 5 min, the supernatant was removed, and the cell pellet was washed with 1 mL PBS, followed by a second centrifugation at 300 g for 5 min. After discarding supernatant, the cells were resuspended in 200 µL PBS. Data was analyzed using Kaluza 2.1 software (Beckman Coulter).

After 72 h, cells were harvested and chemically lysed by the addition of lysis buffer containing 5 mM MgCl_2_, 400 mM NaCl, 50 mM TRIS, and 0.5% (v/v) Tween‐20 (pH 8.0), followed by incubation for 15 min at 37°C with shaking at 120 rpm. Salt Active Nuclease (SAN; ArcticZymes Technologies) was added to a final concentration of 50 U/mL, and the lysate was incubated for 2 h at 37°C with shaking. Clarification was subsequently performed by two consecutive centrifugation steps at 2000 g for 20 min and 4000 g for 10 min at 4°C. The resulting supernatant was collected, and clarified lysate was stored at −80°C until further use. Titer samples collected at the specified time points were clarified using the same protocol.

### 4.4 rAAV DNA Isolation

Clarified rAAV lysate samples were treated with recombinant DNase I (Roche) at a final concentration of 100 U/mL and incubated for 30 min at 37°C to remove remaining external host‐ or plasmid DNA. Next, viral capsids were lysed by addition of proteinase K (Thermo Scientific) to a final concentration of 1 mg/mL in the presence of 0.5% (w/v) SDS (Thermo Scientific), and the samples were incubated for 30 min at 37°C, followed by incubation for 20 min at 95°C. Extracted viral DNA was diluted 1:50 in TE buffer (pH 8.0, 10 mM Tris‐HCl, 0.1 mM EDTA; Thermo Scientific) and stored at −20°C until used for digital droplet PCR (ddPCR).

### 4.5 Digital Droplet PCR for rAAV Genome Quantification

Viral genome titers were determined by ddPCR using the Bio‐Rad QX 200 system (QX 200 Droplet Generator, C 1000 Touch Thermal Cycler, and QX 200 Droplet Reader; Bio‐Rad Laboratories), based on the protocol described elsewhere with minor changes [74]. In detail, isolated rAAV DNA was quantified using a probe‐based ddPCR assay with primer‐probe sets targeting the GFP transgene and the inverted terminal repeat sequences (ITR) of the viral genome (Table 1). Each ddPCR reaction was prepared in a final volume of 22 µL, consisting of 11 µL ddPCR Supermix for Probes (Bio‐Rad Laboratories), primer, and probe sets for GFP and ITR at final concentrations of 0.25 µM each, 250 U/mL MspI restriction enzyme, and nuclease‐free water. For each reaction, 5.5 µL of pre‐diluted DNA in nuclease‐free water was added. Droplet generation, thermal cycling, and droplet reading were performed according to the manufacturer's instructions. For each biological replicate, two independent DNA isolations were analyzed, and each isolation was measured at two different dilutions to ensure accurate quantification within the dynamic range of the assay. Only measurements with a relative standard deviation (RSD) of less than 25% across technical replicates were considered acceptable for analysis. Data evaluation and absolute quantification were performed using QX Manager Software, Standard Edition (Bio‐Rad Laboratories).

**TABLE 1:** Primer and probe sequences used for ddPCR quantification of rAAV genomes.

Target	Type	Sequence (5’‐3’)
GPF	Forward	GAACCGCATCGAGCTGAA
GPF	Reverse	TGCTTGTCGGCCATGATATAG
ITR	Forward	GGAACCCCTAGTGATGGAGTT
ITR	Reverse	CGGCCTCAGTGAGCGA
GFP	Probe	5’‐6‐FAM‐TTGCCGTCC‐ZEN‐TCCTTGAAGTCGAT‐Iowa Black FQ
ITR	Probe	5’‐HEX‐CACTCCCTC‐ZEN‐TCTGCGCGCTCG‐Iowa Black FQ

### 4.6 Enzyme‐Linked Immunosorbent Assay for rAAV Capsid Quantification

The concentration of rAAV capsids was determined using an AAV8‐specific ELISA kit (Progen Biotechnik GmbH) according to the manufacturer's instructions. Samples were diluted in 1x assay buffer to fall within the recommended quantification range, and absorbance was measured at 450 and 650 nm using a Tecan Spark plate reader (Tecan Trading AG). The concentration of intact viral capsids was calculated using the F‐SOLVER function with a four‐parameter logistic (4PL) curve‐fitting model. Technical sample replication and acceptance criterion were applied as described for ddPCR.

### 4.7 Mass Photometric Assessment of rAAV Genome Packaging

The ratio of filled to total capsids in final rAAV preparations at 72 h, as determined by ddPCR and ELISA, was validated using the orthogonal mass‐based analysis by mass photometry (SamuxMP, Refeyn). Full‐to‐empty ratios obtained by mass photometry were used to calculate volumetric titers based on total capsids measured by ELISA. Prior to analysis, clarified lysates were purified using a bind‐and‐elute resin‐based affinity purification protocol in batch mode with POROS CaptureSelect AAVX Affinity Resin (Thermo Scientific) to achieve the required sample purity. Purification and measurement were performed in independent technical triplicates for each biological replicate. Data were acquired using Refeyn AcquireMP software, and evaluated with Refeyn DiscoverMP software. Analyzed mass photometry data, including peak fitting and detailed peak evaluation, are provided in the supplementary material (Figures S2–S6).

### 4.8 Total RNA Preparation and Sequencing

Total RNA was extracted from samples stored in RNA Shield (Zymo Research) using the Quick‐RNA Miniprep Kit (Zymo Research) according to the manufacturer's instructions. All procedures were carried out under RNase‐free conditions to prevent RNA degradation. The concentration of isolated total RNA was measured using a nanodrop One C spectrophotometer (Thermo Scientific). To minimize potential batch effects arising from RNA isolation, library preparation, or RNA sequencing, samples were randomized prior to RNA isolation using the experDesign R package [75].

The library preparation and sequencing were performed by the Next Generation Sequencing Facility at Vienna BioCenter Core Facilities (VBCF). RNA sequencing libraries were prepared using an rRNA depletion kit (NEB). Libraries were assessed using a Qubit fluorometer (Invitrogen) and a Fragment Analyzer prior to loading onto a 10B XP flowcell and sequencing on a NovaSeq X system (Illumina), configured to generate a minimum of 30 million paired‐end reads with a read length of 150 bp per sample.

### 4.9 RNA Sequencing Reads Processing

Raw read preprocessing was performed using a fully reproducible data analysis pipeline developed with the workflow manager Nextflow [76] and provided by Markus Riedl (manuscript in preparation). The workflow includes all utilized tools, their versions, and configuration parameters and is available at the author's github repository (https://github.com/NBorthLab/nf‐rnaseq).

In detail, raw sequencing reads in FASTQ format were processed using TrimGalore [77, 78] for quality and adapter trimming with automated adapter detection. Low‐quality bases with a Phred score below 20 were removed and reads shorter than 20 bp after trimming were discarded. Read quality was assessed before and after trimming using FastQC [79] and quality metrics were gathered and summarized with Multiqc [80]. Next, trimmed reads were aligned using STAR aligner [81] to a custom‐extended human reference genome (NCBI RefSeq assembly GCF_000001405.40), that additionally included scaffolds derived from human adenovirus 5 (HAdV.5; RefSeq accession AC_000008.1) and sequences from transfected plasmids. Gene‐level read quantification was performed using the feature counts utility from Subread [82, 83].

### 4.10 Bioinformatic Analysis

Read counts generated by feature counts were imported into R v4.5.1 [84]. Prior to downstream analysis, genes with fewer than 10 reads in at least four samples were filtered, following recommendations in the standard DESeq2 workflow [85]. Regularized logarithm (rlog) or variance‐stabilizing (vst) transformation was applied to filtered gene counts for data exploration using the rlog and vst functions from DESeq2. Both methods perform library size normalization using the median‐of‐ratios approach. Principal component analysis (PCA) was then conducted on the complete set of filtered genes using the plotPCA function from DESeq2 to assess global variance structure and relationship between conditions. Time‐course expression profiles for each condition were generated by specifying contrasts of each sampling timepoint against the reference time point prior to transfection (0 h). The lfcShrink function from DESeq2 with the adaptive shrinkage method (ashr) [86] was used to reduce the influence of lowly expressed genes on log2 fold‐change estimations. Gene set enrichment analysis was performed using the fgsea package [87] and hallmark collection gene sets for human retrieved from MSigDb [88]. Genes were ranked by their log2FC derived from the DESeq2 analysis. Benjamini‐Hochberg adjusted p‐value with a threshold of < 0.05 was applied to identify significantly enriched gene sets for each pairwise comparison at every timepoint. Result filtering, gene clustering and comparative analysis of time‐course expression profiles were performed in R using custom scripts. Clustering of gene expression trajectories (*k* = 3) was based on Pearson correlation distance (d = 1 – r) applying Ward's minimum variance method (Ward.D2). Spearman's rank correlation coefficient was used to analyze the consistency of expression trends for plasmid‐derived genes across all conditions and time points. The ggcorrplot package [89] was used for the visualization of the correlation matrix. Functional enrichment analysis was carried out using the clusterProfiler package [90] and the Metascape utility [91]. For further analysis of expression profiles over time and validation of the DESeq2 results, the R package SplineOmics [42], which is based on the limma framework [92], was used. Features identified with SplineOmics with a p.ajd. < 0.001 and cumulative travel (cT) >2 were considered as significant. Data visualization was generated using ggplot2 [93] from the tidyverse suite [94], ComplexHeatmap [95], and UpSetR [96].

### 4.11 Statistics

Statistical evaluation of titer and count data was performed in R using the aov function to execute an ordinary one‐way ANOVA. For datasets reaching statistical significance (p‐adj. < 0.05), appropriate post‐hoc analyses were performed using the glht function from the multcomp package [97], applying either Dunnett's post‐hoc test, or Tukey's post‐hoc test depending on the experimental comparison.

### 4.12 Verification of RNA Sequencing by Digital Droplet PCR

Isolated and purified total RNA samples were reverse transcribed to cDNA using the ProtoScript II First Strand cDNA Synthesis Kit (New England Biolabs) with oligo(dT) primers according to the manufacturer protocol. Resulting cDNA was quantitatively analyzed by ddPCR utilizing the QX200 EvaGreen Supermix system (Bio‐Rad Laboratories). Two sampling timepoints, 0 h (pre‐transfection) and 48 h post‐transfection were measured in three biological replicates for all five cell lines. Primer pairs for the quantification of housekeeping genes (GAPDH and ACTB) and target genes (STAT1, ISG15, RIG‐I, and IRF1) were acquired from Integrated DNA Technologies (IDT). Primer specificity was verified by PCR and agarose gel electrophoresis prior to ddPCR verification experiments. Samples were analyzed using appropriate cDNA concentrations to stay within the quantitative range of the assay for each target gene. Primer sequences are listed in Table 2. Detailed results are provided in the (Tables S5 and S6; Figures S9–S11).

**TABLE 2:** Primer sequences used for ddPCR quantification of cDNA target genes.

Target	Type	Sequence (5’‐3’)
GAPDH	Forward	GGAAGGTGAAGGTCGGAGTC
GAPDH	Reverse	ATGAAGGGGTCATTGATGGCA
ACTB	Forward	ACCTTCTACAATGAGCTGCG
ACTB	Reverse	CCTGGATAGCAACGTACATGG
STAT1	Forward	ATGGCAGTCTGGCGGCTGAATT
STAT1	Reverse	CCAAACCAGGCTGGCACAATTG
ISG15	Forward	TACAGGAGCTTGTGCCGTG
ISG15	Reverse	GACACCTGGAATTCGTTGCC
RIG‐I	Forward	TGTCCACCTTCAGAAGTGTCT
RIG‐I	Reverse	AGCAGGCAAAGCAAGCTCTA
IRF1	Forward	GAGGAGGTGAAAGACCAGAGCA
IRF1	Reverse	TAGCATCTCGGCTGGACTTCGA

## Author Contributions

**Georg Smesnik**: Conceptualization, writing – original draft, formal analysis, investigation, data curation, visualization. **Nikolaus Virgolini**: Conceptualization, investigation, writing – review and editing. **Astrid Dürauer**: Conceptualization, funding acquisition, project administration, writing – review and editing. **Nicole Borth**: Conceptualization, supervision, writing – review and editing.

## Conflicts of Interest

The authors declare no conflicts of interest.

## Supporting information




**Supporting File 1**: biot70282‐sup‐0001‐SuppMat.docx.

**Supporting File 2**: biot70282‐sup‐0002‐TableS7_ORA_Gene_lists.xlsx.

## Data Availability

The data that supports the findings of this study are available in the supplementary material of this article.
