# Supplementary material for: Comparative Transcriptomic Profiling Reveals Differences in Initiation of Antiviral Response in Low rAAV Producing HEK293 Suspension Cells
Source: Biotechnol J. 2026 Jul 9;21(7):e70282. doi: 10.1002/biot.70282 (PMC13347752; doi:10.1002/biot.70282)
Supplement: Supplementary file 1 — Supporting File 1: biot70282‐sup‐0001‐SuppMat.docx. [file BIOT-21-e70282-s002.docx]

# Supplementary Material

Content of supplementary material

1. Cell growth and viability prior and during rAAV production
2. Analyzed mass photometry data
3. Transfection efficiency assessment
4. Gene set enrichment analysis
5. Correlation of plasmid gene expression
6. Clustering of top differentially expressed genes
7. Comparative B-spline analysis
8. Statistics to BalCD-specific response genes
9. E1A and E1B gene expression kinetics
10. Additional pathway analysis
11. RNA-sequencing verification by ddPCR
12. Additional information

## Growth and viability prior and during rAAV production

HEK293 cells were monitored for growth and viability following recovery from the thawing procedure to ensure the cultures were suitable for the experiment. Prior to transient rAAV production, cultures were expanded through sequential passages to achieve the required culture volumes and cell numbers. Next, four biological replicates of each cell line were seeded at a starting volume of 75 mL and a viable cell density of 2 × 10⁶ cells/mL, followed by transfection with PEI and a triple-plasmid transfection mixture as described in the Materials and Methods section. Cultures were sampled at the indicated timepoints post-transfection (6, 18, 24, 30, 42, 48, and 72 h), and viable cell density was determined using the trypan blue exclusion method with an automated ViCell XR device.


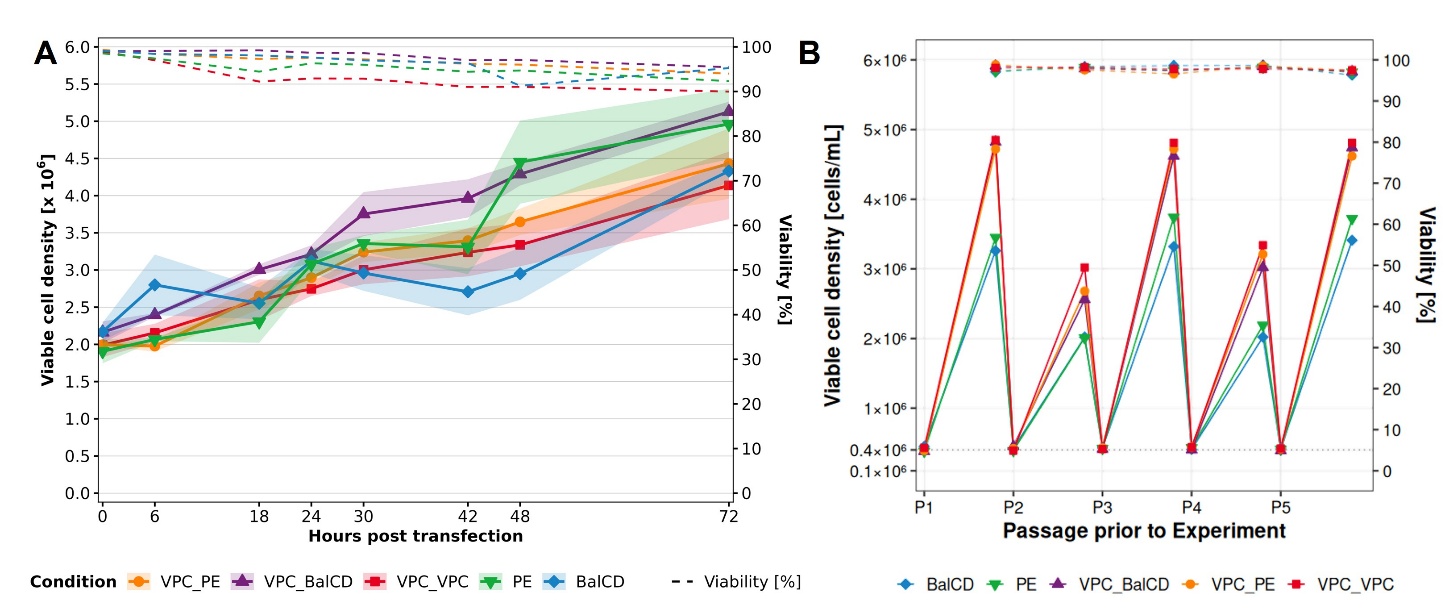


Figure S1: Growth and viability (A) during and (B) before rAAV production. (A) Specific growth rates and viability profiles over the course of transient rAAV production for five conditions: VPC cells in VPC medium (VPC_VPC), VPC cells adapted to BalCD medium (VPC_BalCD), VPC adapted to PE medium (VPC_PE), In-house generated suspension cell line cultivated in PE medium (PE), In-house generated suspension cell line cultivated in BalCD medium(BalCD). Data points represent mean viable cell density from four biological replicates, with shaded areas indicating standard deviation. Dashed lines denote mean viability for each culture. (B) Cell growth and viability prior to culture expansion and rAAV production. Cultures were monitored across alternating passaging intervals of four days (P1–P2; P3–P4) and three days (P2–P3; P4–P5). The dotted grey line indicates the target seeding density of 0.4 × 10⁶ viable cells/mL. Dashed lines denote viability for each culture. (A, B) Colors and shapes correspond to the respective conditions.

## Analyzed mass photometry data

Mass photometry was applied as an orthogonal method to ddPCR and ELISA to assess the ratio of filled to total capsids in final rAAV harvests collected 72 h post-transfection. Samples were prepared prior to analysis to achieve the required purity, as described in the Materials and Methods section. For each biological replicate, three independent purifications were analyzed, except for VPC cells cultured in PE medium, for which preparations of a single biological replicate yielded insufficient material for measurement. Peak integration was performed using Refeyn DiscoverMP software with Gaussian distribution–based peak fitting. Obtained full-to-empty ratios were used to calculate volumetric titers based on total capsids measured by ELISA.


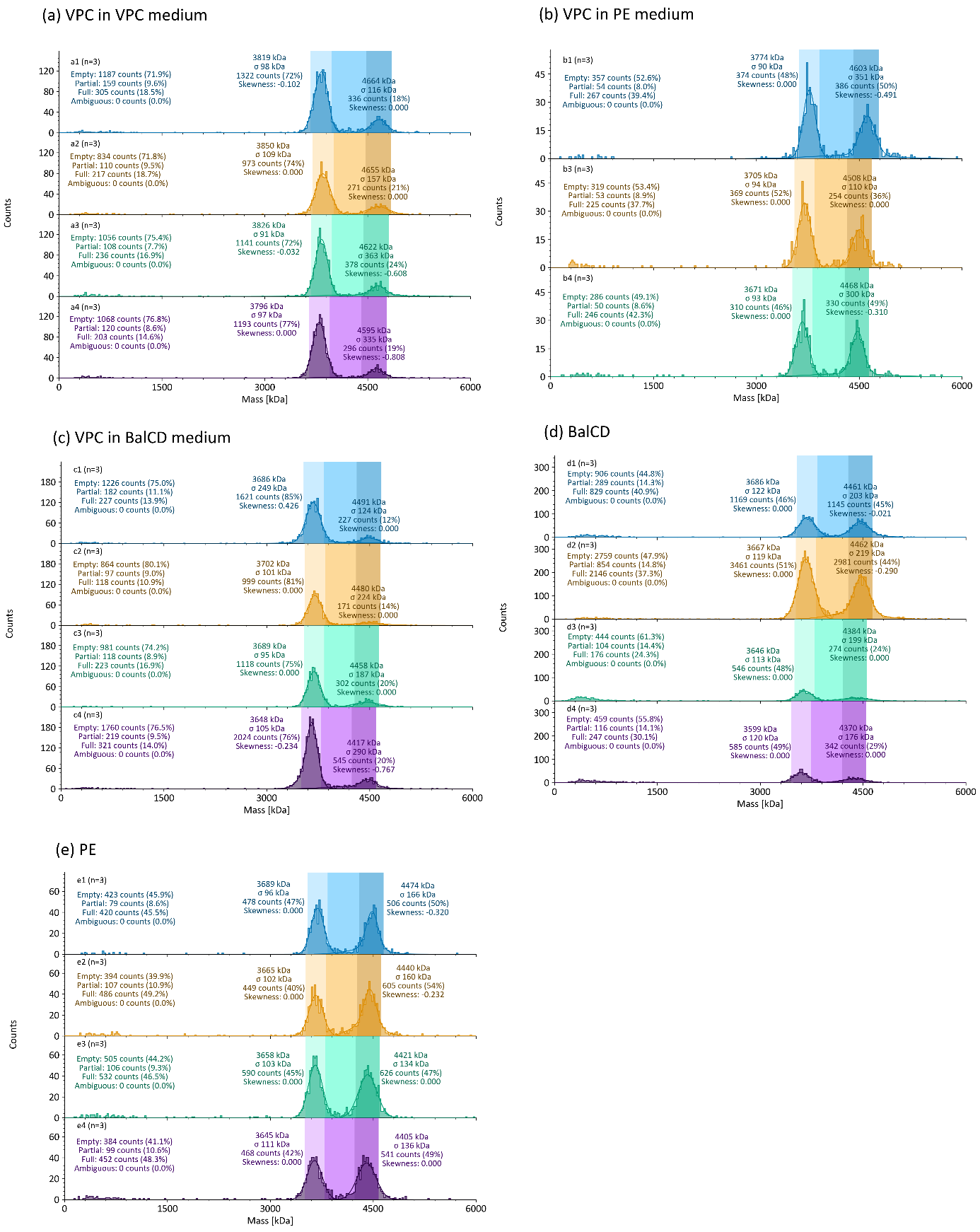


Figure S2: Mass photometry analysis of rAAV samples. Panels (a-e) show mass photometry measurements of individual biological replicates following additional purification. Histogram colors separate mean distributions from three measurements per biological replicate. Peak annotations indicate number of measured particles, estimated particle mass (kDa), and Gaussian skewness. Peaks are classified as empty, partial, full or ambiguous, as indicated to the left of each histogram.

## Transfection efficiency assessment


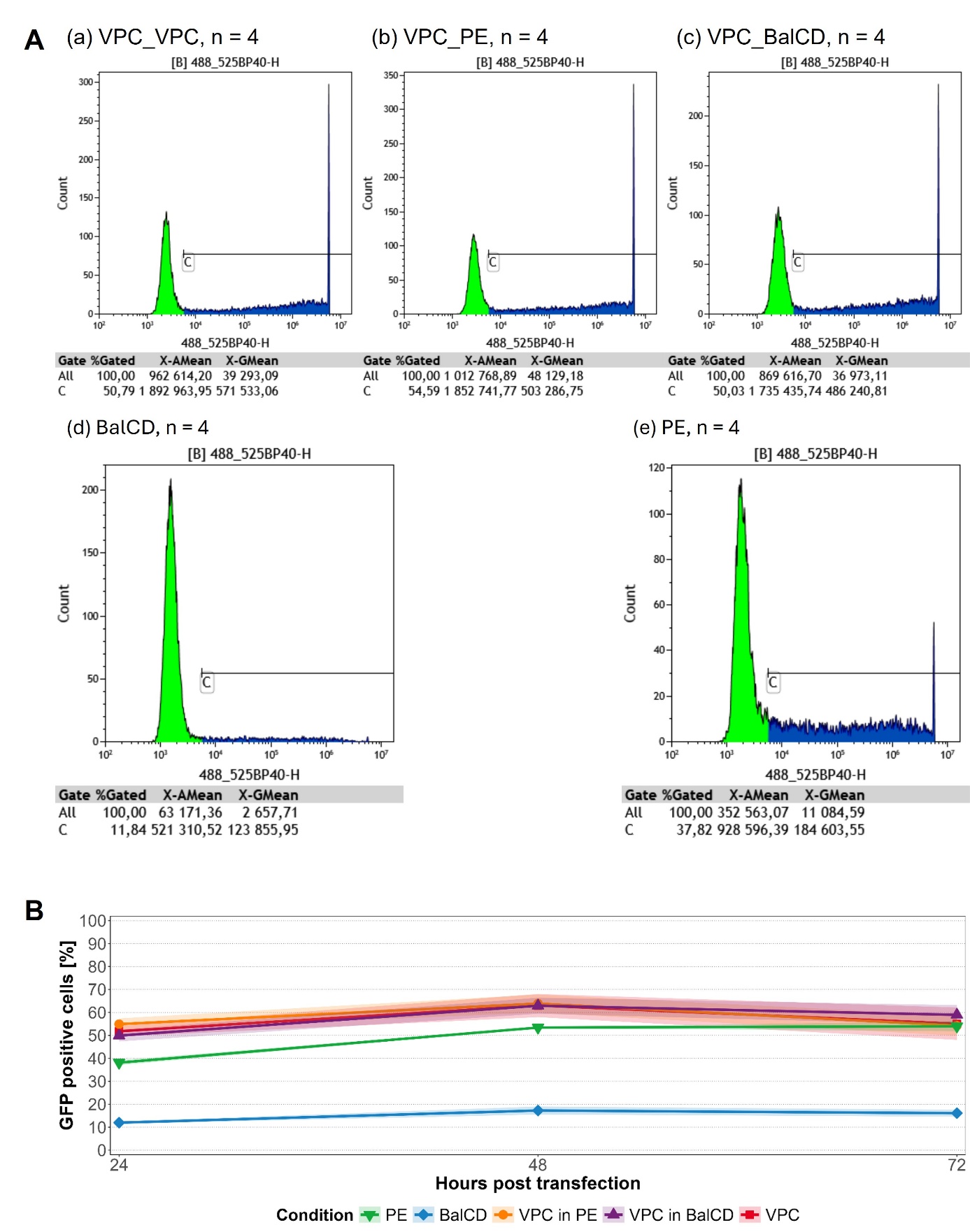


Figure S3: Transfection efficiency assessment across cell lines. (A) Flow cytometry histograms of GFP expression at 24 h post-transfection for four biological replicates per indicated cell line (a–e). Green peaks denote GFP-negative cells, while blue distributions represent the GFP-positive population. The percentage of GFP-positive cells, as well as the arithmetic and geometric mean fluorescence intensities, are indicated below each histogram. (B) Quantification of GFP-positive cells over time (24, 48, and 72 h post-transfection). Data points represent mean values from four biological replicates, with shaded areas indicating standard deviation. Colors and shapes correspond to conditions.

## Gene set enrichment analysis

To investigate distinct expression profile of the low-producing BalCD cell line, a Gene Set Enrichment Analysis (GSEA) was performed using the Hallmark gene set collection from the Molecular Signatures Database (MSigDb). Pairwise contrasts were conducted between BalCD and each individual high-producing condition (BalCD vs. VPC_VPC, BalCD vs. VPC_PE, BalCD vs. VPC_BalCD, BalCD vs. PE). To capture the consistent signature of the low producer, the resulting Normalized Enrichment Scores (NES) are presented here as average across these contrasts, provided the enrichment followed the same direction in all comparisons. In this setup, a positive NES indicates an enrichment or up-regulation of a pathway in BalCD relative to the high-producing cell lines, whereas a negative NES reflects lower expression levels in the low producer.

Pathways were ranked based on their NES at 30 h post-transfection as this timepoint represented the maximum of enrichment of Interferon type I and type II pathways. The NES was calculated for all timepoints (0-72 h) to track the fate of the observed pathways and investigate for cell line specific signatures in BalCD at timepoint 0 h prior to transfection.

Table S1: Summary of temporal Gene Set Enrichment Analysis (GSEA) of the Low-Producer BalCD. The table displays averaged Normalized Enrichment Scores (NES) derived from pairwise contrasts between BalCD and high-producing cell lines. Scores were averaged across all comparisons where the direction of enrichment was consistent. In this setup, a positive NES indicates an enrichment or up-regulation of the pathway in the low-producing BalCD relative to the high-producers, while a negative NES reflects lower expression levels in BalCD. Listed pathways represent the top-ranking hallmarks significantly enriched at the 30 h post-transfection reference point (ranked descending by NES).

## Correlation of plasmid gene expression

To assess the transcriptional relationship and kinetic synchrony of representative plasmid-derived genes across all cell lines and time points, a correlation analysis was performed. Transcript counts were log-transformed and Z-score scaled to account for varying expression magnitudes between individual genes. Pairwise correlations were calculated using the Spearman’s rank correlation coefficient (ρ) to evaluate the consistency of expression trends across all conditions and time points.


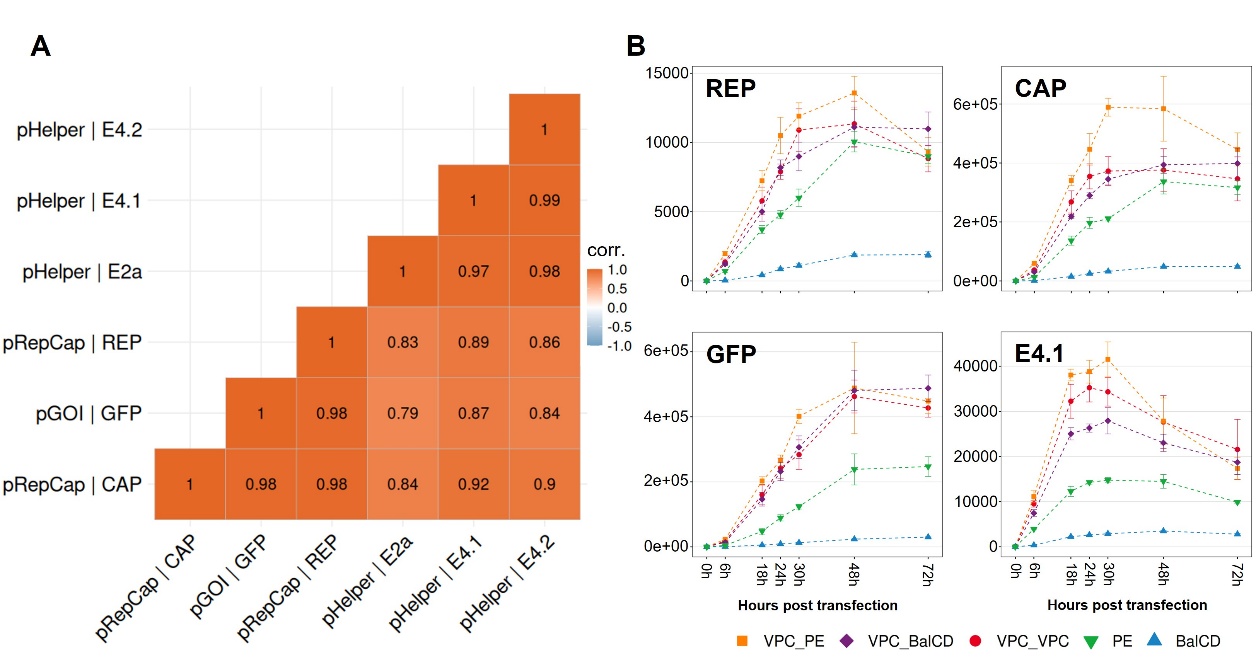


Figure S4: Expression correlation and kinetics of plasmid-derived genes. (A) Spearman correlation matrix of a representative subset of plasmid-derived transcripts across all cell lines (conditions) and time points. The matrix displays Spearman’s rank correlation coefficients ( -1 to 1), reflecting the degree of synchrony in expression dynamics between genes from the three transfected plasmids (pRepCap, pHelper, pGOI). Gene names E4.1 and E4.2 represent two distinct open reading frames (ORFs) of the adenoviral E4 helper gene. (B) Kinetic profiles of selected plasmid-derived genes (REP, CAP, GFP, E4.1). Data points represent the mean of normalized counts (median-of-ratios method) at each indicated time point, with colors and shapes corresponding to conditions. Error bars indicate standard deviation among four biological replicates. Dashed lines illustrate expression trajectories over the time course.

## Clustering of top differentially expressed genes

Pearson correlation distance combined with Ward’s minimum variance method was used to cluster differentially expressed genes based on their time-course expression profiles within each cell line into three distinct clusters. Gene subsets derived from these clusters were subjected to gene ontology and pathway enrichment analysis, and the results were compared to identify overlapping biological functions among the high-producing cell lines. Shown here are the overlapping terms identified in Cluster 1, along with functional enrichments for Cluster 2. Cluster 3 contained insufficient gene numbers to yield statistically significant enrichments.


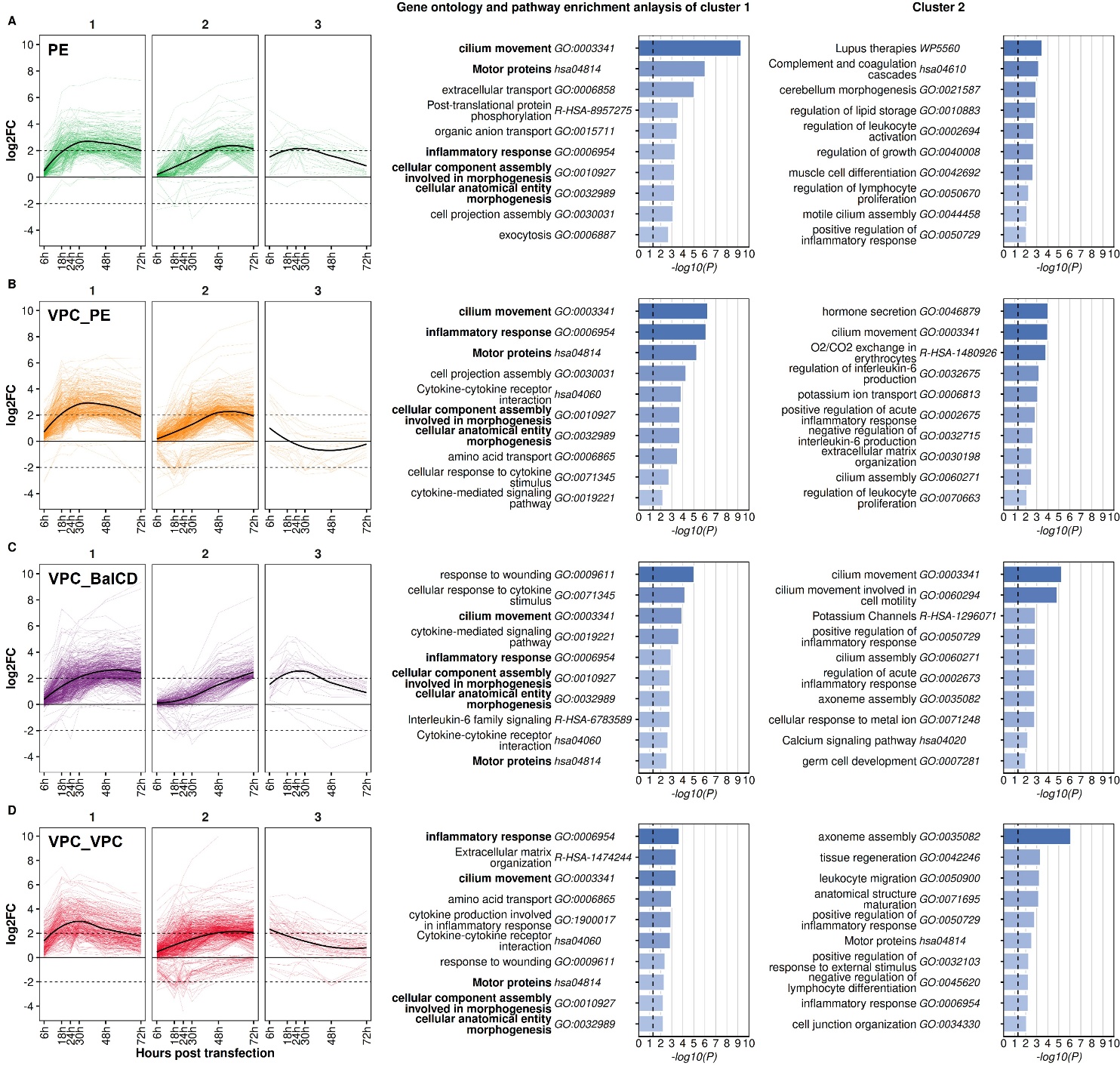


Figure S5: Clustering and enrichment analysis of top differential expressed genes over time. (A–D) Line plots depict log2FC relative to baseline expression at 0 h (pre-transfection) across the production time course for each condition. Gene expression trajectories are grouped into three clusters (panels 1–3) based on Pearson correlation distance using Ward’s minimum variance method, with solid black lines indicating cluster centroids. Right panels show gene ontology and pathway enrichment analysis for genes in cluster 1; bar length represents −log10 p-value, and the vertical dashed line denotes the significance threshold.

## Comparative B-spline analysis

B-spline analysis, implemented using the SplineOmics R package and based on the limma framework, was performed to identify gene expression trajectories over the time course of transient rAAV production within each condition. This approach enables the identification of statistically significant spline shapes, their clustering, and comparison across conditions. Changes in gene expression were quantified as absolute cumulative travel (cT), which was used as an additional filtering criterion to select splines exhibiting a significant temporal pattern and a cumulative travel > 2. The resulting subset was used for comparative analysis to identify genes that were either shared among high-producing cell lines or exclusive to the low-producing BalCD line. These gene sets were subsequently subjected to gene ontology and pathway enrichment analysis.


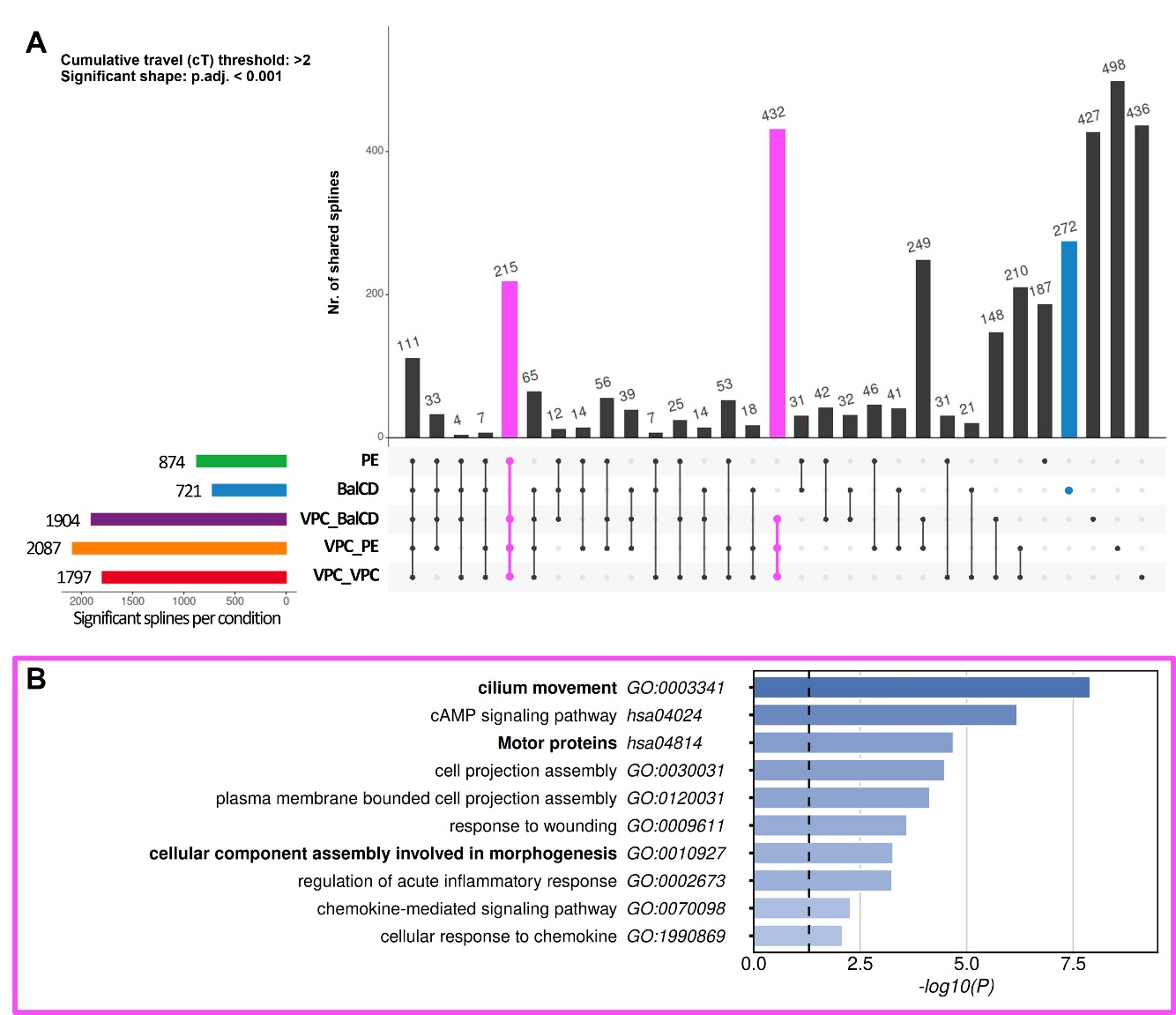


Figure S6: (A) UpSet plot showing all combinations of shared and exclusive significant splines passing the pre-filtering criteria as annotated in the upper-left panel. Dots and connecting lines in the matrix show the splines shared between samples, with their number shown by the corresponding black, pink or blue bars respectively. Combinations shared among high-producing cell lines are highlighted in pink, while splines exclusive to BalCD are highlighted in blue. Colored bars on the left indicate the total number of significant splines per condition subjected to the analysis. (B) Gene ontology and pathway enrichment analysis of shared splines in high-producing cell lines (pink bars). Bar length represents the -log10 p-value, the vertical dashed line indicates the significance threshold. Enrichment results for the BalCD-exclusive spline set are shown in Figure 4A.

## Statistics to BalCD-specific response genes

Statistical results of pairwise differential gene expression analysis at 48 h post-transfection (DESeq2) are shown for selected genes involved in the rapid antiviral response, which are highly upregulated in BalCD compared to high-producing cell lines.

Table S2: Pairwise expression comparisons relative to BalCD at 48h post-transfection. Shown are log2FC and adjusted p-values for the indicated genes of interest in BalCD versus each reference cell line. Log2FC >1 are highlighted in green; p-adj values exceeding the significant threshold of <0.05 are highlighted in red.

## E1A and E1B gene expression kinetics

Here we provide supplementary data on the expression kinetics of the HEK293 integrated adenoviral helper genes E1A and E1B55k. Figure S7 illustrates the relative expression differences between conditions at 24 h, 48 h, and 72 h post-transfection, normalized to the low-producer BalCD. Notably, increased basal expression levels for both genes were observed in BalCD already at 0 h (pre-transfection) and remained higher throughout the production period compared to all other conditions. To investigate differences in expression dynamics, we evaluated the rates of change (deltas) of normalized counts across three time intervals: (Δ 0 – 30 h; Δ 24 – 72 h, Δ 30 – 72h). To identify divergent transcriptional trends, a one-way ANOVA followed by Tukey’s post-hoc test was performed on these calculated deltas. Detailed statistical parameters, including estimates, confidence intervals, and adjusted p-values, are provided in Table S3 and Table S4.


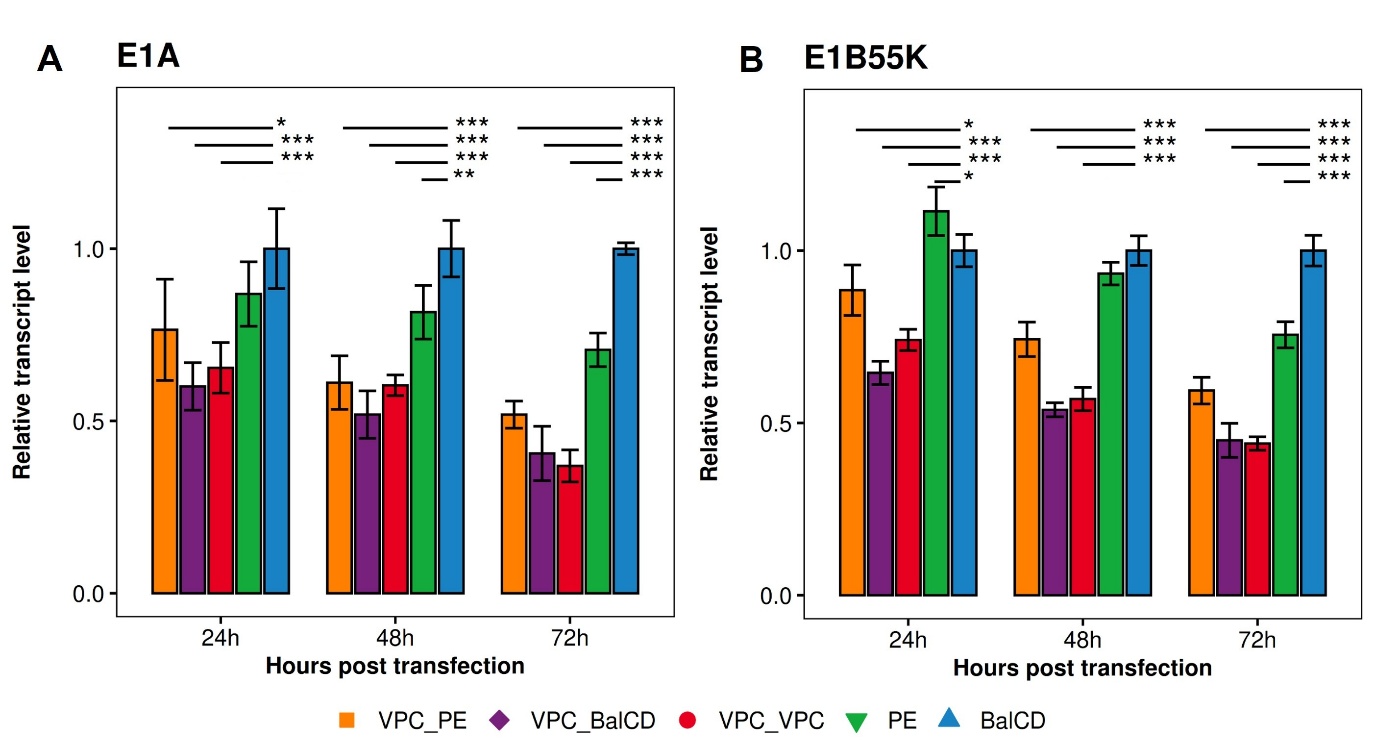


Figure S7: Relative transcript levels of (A) E1A and (B) E1B55K. Bar plots show transcript levels of each cell line relative to BalCD at the indicated timepoints. Error bars indicate standard deviation among four biological replicates. Horizontal lines and asterisks indicate significant differences by one-way ANOVA with Dunnett’s post hoc test (p-adj: *** <0.001, ** <0.01, * <0.05). Gene symbols are annotated on top of each panel.

Table S3: Statistical evaluation of E1A expression dynamics across specific time intervals. The table presents the results of a one-way ANOVA followed by Tukey’s post-hoc test performed on the rates of change of normalized counts at the indicated time periods. For each contrast, the estimate (mean difference in deltas), 95% confidence intervals (conf. low, conf. high) and the adjusted p-value are provided. Significant differences (adj-p. <0.05) are highlighted in red.

Table S4: Statistical evaluation of E1B expression dynamics across specific time intervals. The table presents the results of a one-way ANOVA followed by Tukey’s post-hoc test performed on the rates of change of normalized counts at the indicated time periods. For each contrast, the estimate (mean difference in deltas), 95% confidence intervals (conf. low, conf. high) and the adjusted p-value are provided. Significant differences (adj-p. <0.05) are highlighted in red.

## Additional pathway analysis


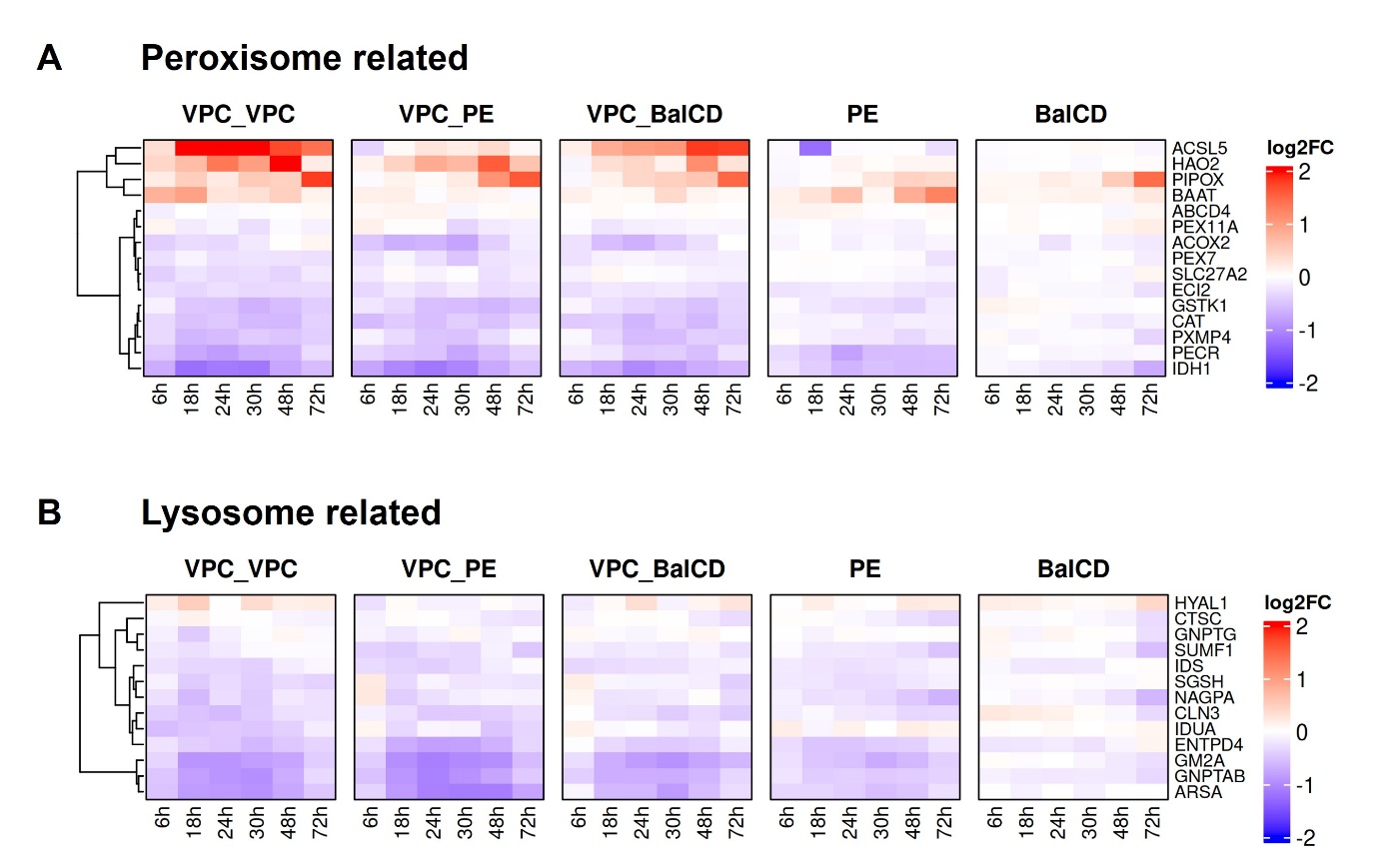


Figure S8: (A) Peroxisome and (B) lysosome related genes shown on heatmaps of time-course expression profiles (log2FC relative to 0 h) across all conditions. Row-wise hierarchical clustering of left panel was used to determine gene order, gene symbols are annotated on the right. Color scale represents log2FC ranging from -2 (blue, decreased) to 2 (red, increased), with white indicating no change.

## RNA-sequencing verification by ddPCR

RNA sequencing results were verified by digital droplet PCR (ddPCR) measurements of selected target genes involved in the rapid antiviral response in BalCD, including RIG-I, IRF1, STAT1 and ISG15. GAPDH and ACTB were used as housekeeping genes.

Prior to ddPCR analysis, purified total RNA samples previously used for RNA sequencing were reverse-transcribed into cDNA as described in the Material and Methods section. Appropriate cDNA concentrations were determined based on the expected expression levels of the respective target gene to allow measurements within the quantitative range of the assay. Three biological replicates for each cell line were analyzed at two sampling timepoints, 0 h (pre-transfection) and 48 h post-transfection. Expression levels were quantified as copies per ng cDNA, as shown in Table S2. Fold changes in expression between 0 h and 48 h are provided in Table S3. It has to be noted that fold change calculations based on very low initial values are highly limited in accuracy. Representative ddPCR raw data showing a single replicate per timepoint and cell line for the indicated targets are presented in Figure S7. Quantified ddPCR results for all cell lines and target genes are shown and compared with RNA-seq raw read counts in Figure S8 and S9.

Overall, the ddPCR verification experiment confirmed the exclusive expression patterns in BalCD for all analyzed target genes.

Table S5: Results of ddPCR measurements expressed as copies per nanogram of cDNA for each target gene at 0 h (pre-transfection) and 48 h post-transfection.

| Target Gene | **VPC_VPC** | | **VPC_PE** | | **VPC_BalCD** | | **PE** | | **BalCD** | |
| --- | --- | --- | --- | --- | --- | --- | --- | --- | --- | --- |
|  | 0h | 48h | 0h | 48h | 0h | 48h | 0h | 48h | 0h | 48h |
| GAPDH | 16592 | 20273 | 18393 | 17208 | 35989 | 25726 | 11639 | 13293 | 7894 | 17526 |
| ACTB | 23565 | 24913 | 22726 | 17769 | 34410 | 28533 | 13481 | 12460 | 10372 | 20994 |
| STAT1 | 276 | 320 | 309 | 257 | 479 | 446 | 162 | 223 | 126 | 3392 |
| ISG15 | 34 | 160 | 47 | 157 | 58 | 130 | 11 | 97 | 10 | 6658 |
| RIG-I | 22 | 30 | 27 | 30 | 28 | 39 | 17 | 35 | 17 | 1189 |
| IRF1 | 23 | 29 | 31 | 30 | 43 | 50 | 12 | 18 | 13 | 79 |

Table S6: Results of ddPCR measurements expressed as fold changes between 0h (pre-transfection) and 48h post-transfection for each target gene.

| Target Gene | **VPC_VPC** | **VPC_PE** | **VPC_BalCD** | **PE** | **BalCD** |
| --- | --- | --- | --- | --- | --- |
|  | 48h / 0h | 48h / 0h | 48h / 0h | 48h / 0h | 48h / 0h |
| GAPDH | 1.22 | 0.94 | 0.71 | 1.14 | 2.22 |
| ACTB | 1.06 | 0.78 | 0.83 | 0.92 | 2.02 |
| STAT1 | 1.16 | 0.83 | 0.93 | 1.38 | 26.87 |
| ISG15 | 4.82 | 3.36 | 2.23 | 8.94 | 654.93 |
| RIG-I | 1.36 | 1.11 | 1.38 | 2.03 | 71.75 |
| IRF1 | 1.25 | 0.96 | 1.15 | 1.52 | 6.13 |


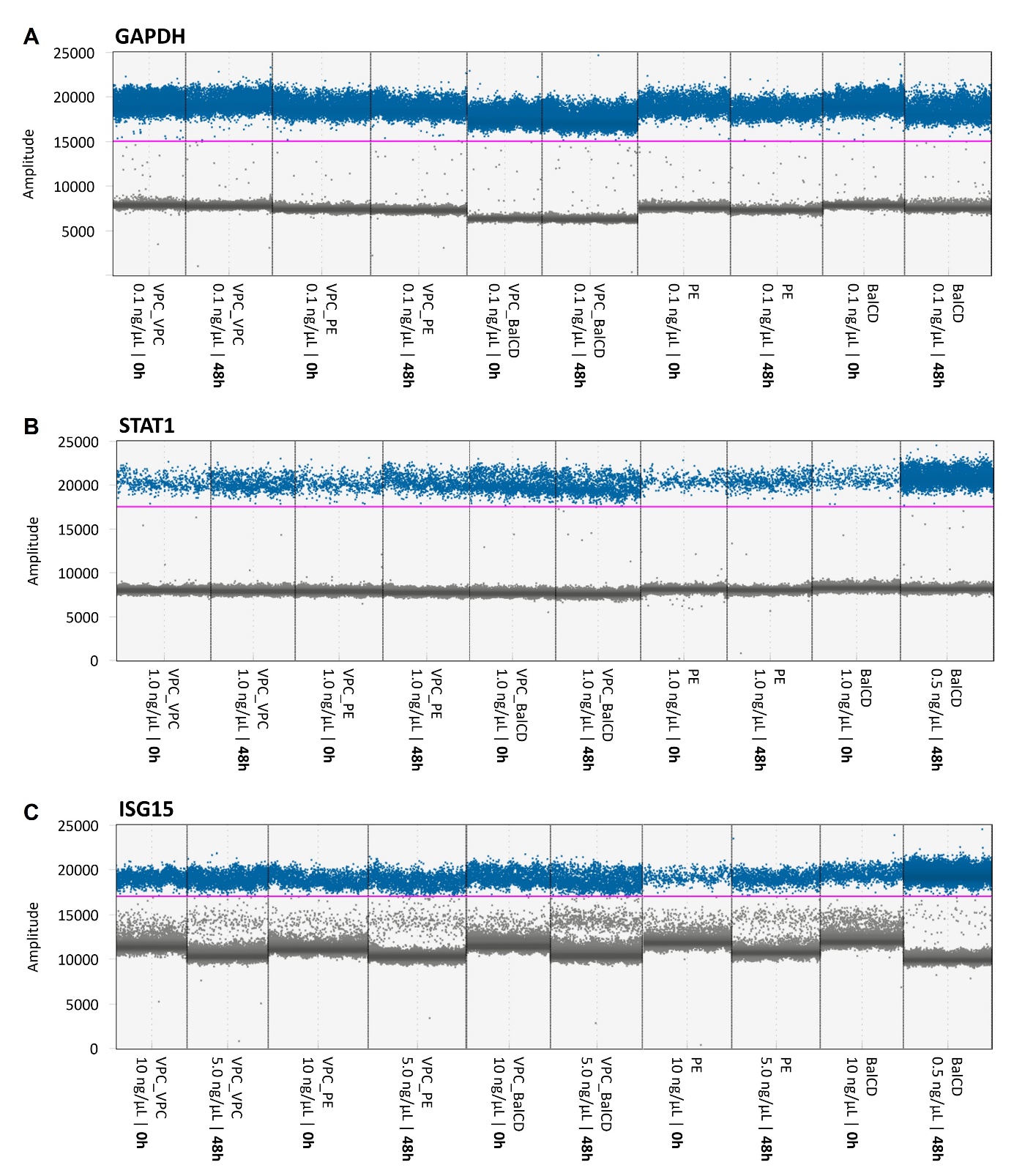


Figure S9: Representative raw ddPCR data showing a single replicate per cell line and timepoint of the indicated target gene. The scatter plot displays fluorescence amplitude for individual droplets, distinguishing positive droplets (blue), which contain amplified target sequences, from negative droplets (grey), which lack amplified target. The purple line indicates the manually defined threshold used to classify droplets as positive or negative. To remain within the quantitative range of the assay, different cDNA concentrations were used depending on the expected target expression levels. Final values were calculated based on 1 ng/µL cDNA from three biological replicates and are reported in Table S2.


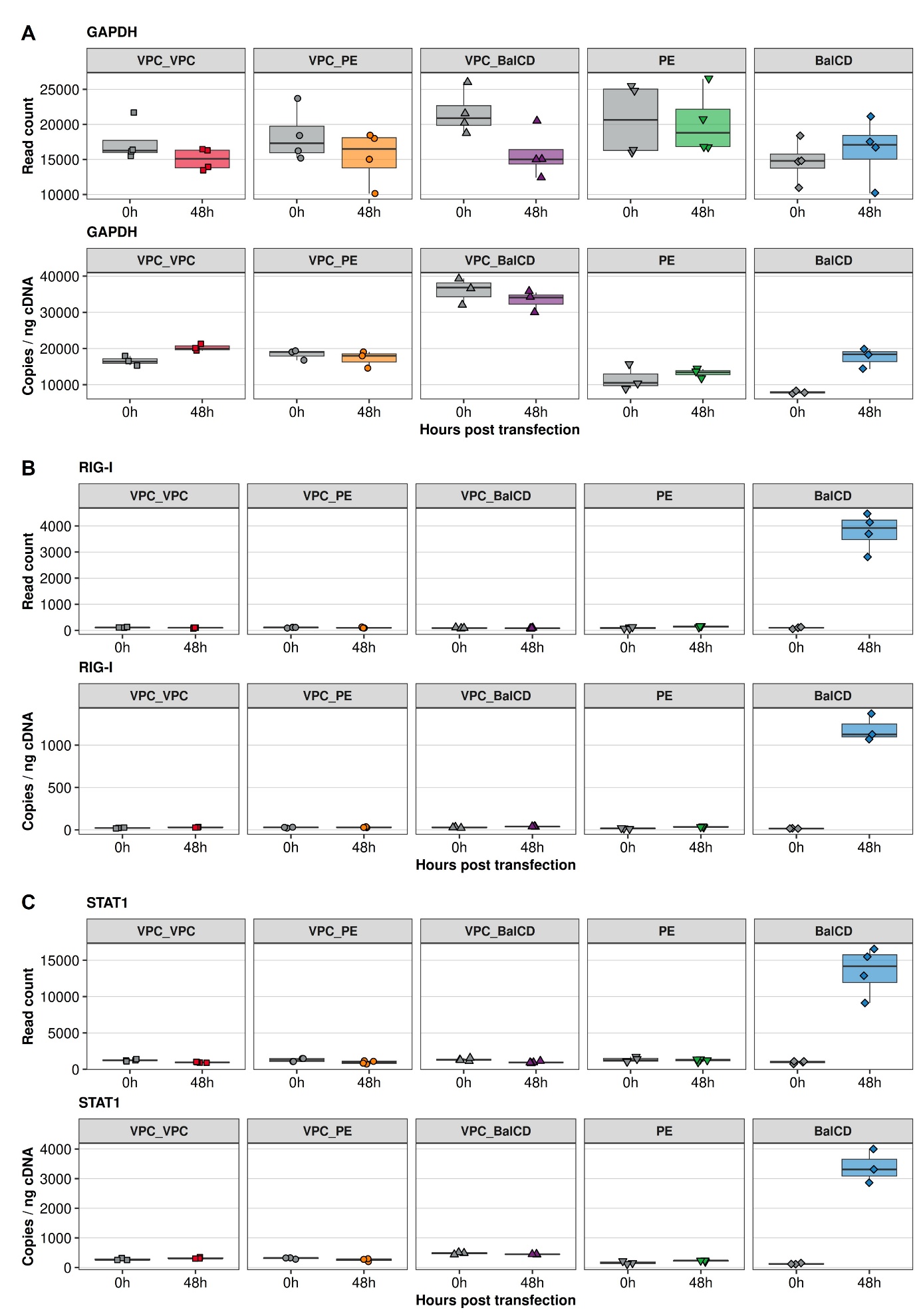


Figure S10: Comparison of RNA-seq and ddPCR expression measurements for selected target genes. Boxplots show expression levels of GAPDH (A), RIG-I (B), and STAT1 (C) at 0h (pre-transfection) and 48h post-transfection across all indicated cell lines. RNA-seq data are shown as raw read counts (upper panels), ddPCR results are expressed as copies per ng cDNA (lower panels). Idividual data points represent biological replicates (RNA-seq: n=4; ddPCR: n=3).


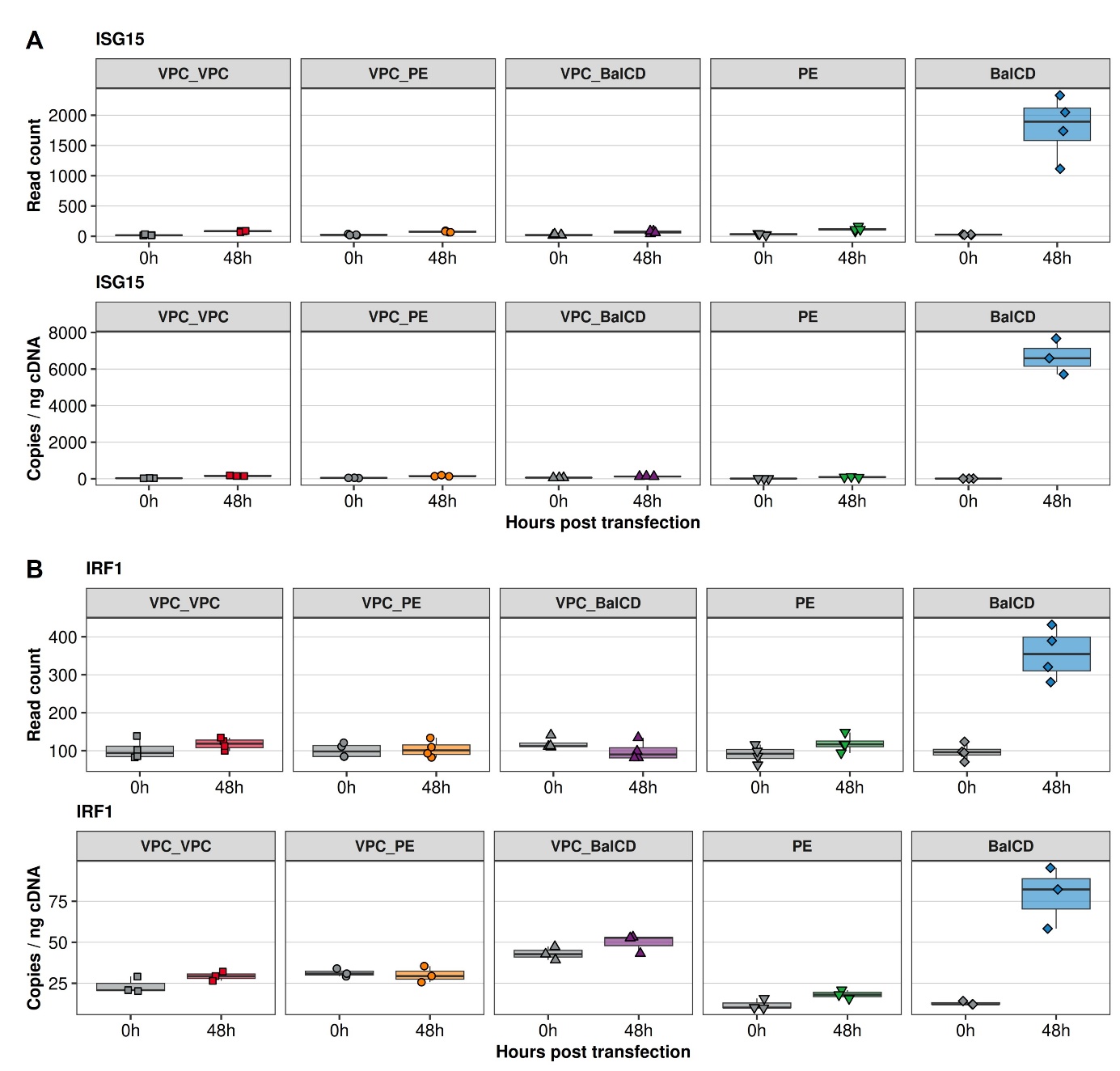


Figure S11: Comparison of RNA-seq and ddPCR expression measurements for selected target genes. Boxplots show expression levels of ISG15 (A) and IRF1 (B) at 0h (pre-transfection) and 48h post-transfection across all indicated cell lines. RNA-seq data are shown as raw read counts (upper panels), ddPCR results are expressed as copies per ng cDNA (lower panels). Idividual data points represent biological replicates (RNA-seq: n=4; ddPCR: n=3).

## Additional information

Table S7 provides gene lists generated from clustering and comparative B-spline analysis that were subjected to gene ontology and pathway enrichment analysis.

Table S7: TableS7_ORA_Gene_lists.xlsx
